# Testicular Expression of Vitamin-D Receptor and Vitamin-D-Metabolizing Enzymes (CYP2R1, CYP27A1, and CYP27B1) in Normal and Cryptorchid Horses

**DOI:** 10.3390/ani16193046

**Published:** 2026-09-28

**Authors:** Maria Albrizio, Mario Cinone, Antonio Ciro Guaricci, Luca Lacitignola, Edmondo Ceci, Salvatore Desantis

**Affiliations:** 1 Department of Regenerative and Precision Medicine-Jonian Area (DiMePRe-J), University of Bari Aldo Moro, 70121 Bari, Italy; mario.cinone@uniba.it (M.C.); antoniociro.guaricci@uniba.it (A.C.G.); luca.lacitignola@uniba.it (L.L.); salvatore.desantis@uniba.it (S.D.); 2 Department of Veterinary Medicine, University of Bari Aldo Moro, 70121 Bari, Italy; edmondo.ceci@uniba.it

**Keywords:** retained testes, stallion, vitamin D, RNA, VDR, hydroxylase, CYP, immunohistochemistry, reproduction

## Abstract

Vitamin D (VD) is required for testis activity being a key regulator of calcium and testosterone, both essential for spermatogenesis and sperm maturation. VD is activated by a metabolizing machinery of enzymes (CYP2R1, CYP27A1, CYP27B1) and acts throughout its receptor (VDR). Cryptorchidism is a developmental defect caused by the failed scrotal descend of the testicle and can compromise fertility depending on whether one or both testes are retained. Here, we reported the expression and localization of VDR- and VD-activating enzymes in the testis of normal and unilateral cryptorchid equines obtained by Real-Time PCR, Western blotting, and immunohistochemistry. Results demonstrated that cryptorchidism affected gene and protein expression. Leydig cells expressed the highest level of VDR, CYP2R1, CYP27A1, and CYP27B1, whilst CYP27A1 and CYP27B1 were more abundant in spermatids from normal testes compared to scrotal testes of unilateral cryptorchid animals. No different cellular localization of the VD-metabolizing machinery was observed between cryptorchid and scrotal testicles, although some variation in staining intensity was observed. These results provide insights for further studies, required for clarifying VD’s role in equine reproduction.

## Simple Summary

Vitamin D (VD) is required for testis activity being a key regulator of calcium and testosterone, both essential for spermatogenesis and sperm maturation. VD is activated by a metabolizing machinery of enzymes (CYP2R1, CYP27A1, CYP27B1) and acts throughout its receptor (VDR). Cryptorchidism is a developmental defect caused by the failed scrotal descend of the testicle and can compromise fertility depending on whether one or both testes are retained. Here, we reported the expression and localization of VDR- and VD-activating enzymes in the testis of normal and unilateral cryptorchid equines obtained by Real-Time PCR, Western blotting, and immunohistochemistry. Results demonstrated that cryptorchidism affected gene and protein expression. Leydig cells (testosterone-producing cells) expressed the highest level of VDR, CYP2R1, CYP27A1, and CYP27B1, whilst CYP27A1 and CYP27B1 were more abundant in spermatids from normal testes compared to scrotal testes of unilateral cryptorchid animals. No different cellular localization of the VD-metabolizing machinery was observed between cryptorchid and scrotal testicles, although some cell variation in staining intensity was observed. These results provide insights for further studies, required for clarifying VD’s role in equine reproduction.

## 1. Introduction

Cryptorchidism is a developmental defect in both animals and humans caused by failure of the testis to descend into the scrotal sac. In equines, this reproductive defect occurs in 5–8% of subjects [1] and can compromise fertility depending on whether one or both testes are retained in the abdominal cavity, inguinal canal, or in a subcutaneous position. Moreover, abdominal retention strongly affects fertility because spermatogenesis fails to occur at warm temperatures [2]. In general, the incidence of testicular retention in the abdomen is seen in 47–60% in equines, 90% in dogs, and 8% in humans [3]. The etiology of cryptorchidism is still under debate because a variety of genetic (polymorphism in different genes) and environmental risk factors (endocrine disruptors, extreme climate) are recognized as acting on the precise timing and coordination of the physiology of testicular descent [4]. Calcium [5] and testosterone [6] are two main regulators of male fertility. There is a strong relationship between calcium levels and male fertility because this ion is required in the testis for steroidogenesis [7], and spermatogenesis [8], while outside the gonad, is required for sperm motility, hyperactivation, and acrosome reaction [9]. In equines, cryptorchidism compromises fertility and reduces but not abolishes testosterone secretion [10]; furthermore, the residual capacity of the testicle to synthesize testosterone is responsible for undesirable sexual behaviors such as sexual excitement [11].

Vitamin D (VD) is a key regulator of systemic calcium homeostasis [12]. When produced by the skin, VD is inactive [13] and it requires a systemic activation, first in the microsome of the liver by two different 25-hydroxylases belonging to the cytochrome P450 family (CYP2R1 and CYP27A1) and then in the mitochondria of the proximal tubular cells of the kidney, where the 25OH-VD is 1α-hydroxylated to the hormonally active 1,25-dihydroxy-VD (1,25(OH)2VD) by CYP27B1 (1α-hydroxylase) [14]. VD acts on target cells by binding to its nuclear receptor (VDR) and heterodimerizing with the retinoid X receptor. The obtained complex behaves as a transcription factor to regulate target genes that contain a VD response element in their promoter. Recently, a VDR integrated in the plasma membrane has been discovered that explains the VD non-genomic action [15] that involves the activation of signaling molecules, the rapid generation of second messengers, and the opening of Ca^2+^ and Cl^−^ channels [16].

The physiology of VD has not been studied in great depth in equines, and the few published studies suggest that VD may act differently for other species. It seems that equine skin is incapable of converting 7-dehydrocholesterol into VD after ultraviolet B-light exposure, so equines rely on their diet as primary sources of ergocalciferol, namely VD_2_ [17]. Furthermore, equids are different from other animal species in absorbing and secreting calcium. In the proximal half of the small intestine, they can absorb a higher proportion of the ion from their diet when compared to ruminants [18], and they show weaker renal reabsorption and a high urinary calcium excretion. In general, plasma calcium level is higher than in other animal species [19] associated with low serum concentrations of VD [20].

In humans [21,22] and in several animal species [23,24], the testicular contribution to VD bioactivation has been demonstrated, as well as the localization of VDR [21], while in the equine, the role of the testis is still questionable because of the peculiar calcium homeostasis.

The testis contribution to VD metabolism has been investigated in equines after castration by Rouge et al. [25], reporting that in this species, the enzymes involved in VD activation are very low or not expressed. For this reason, the equines were considered a unique model in comparative endocrinology. Nevertheless, in recent years, the role of VD as an anti-cancer agent has been highlighted [26]; thus, it seems interesting to investigate the presence of VDR and VD-metabolizing enzymes in equine testes to provide evidence on the recruitment of this organ as a VD-activating site, particularly in subjects that have a high risk of developing abnormal proliferation in the gonad, as in cryptorchidism [27].

In this study, we evaluated the gene and the proteomic expression of VDR and of the three VD-metabolizing enzymes (CYP2R1, CYP27A1, and CYP27B1), as well as their cytological localization in the testis of normal and unilateral cryptorchid equines. This was to provide new insights into the molecular and physiological changes induced in the testis by cryptorchidism.

## 2. Materials and Methods

### 2.1. Experimental Design

Because the aim of the study was to evaluate, in the equine, the effects of cryptorchidism on testicular expression of VDR and three VD-metabolizing enzymes, a group of unilateral cryptorchid subjects under clinical care at the “Veterinary surgical section” of the Department of Precision and Regenerative Medicine-Jonian Area (DiMePRe-J) of the University of Bari-Aldo Moro, were enrolled.

Eighteen animals were studied, five normal (employed as controls) and thirteen cryptorchids that were split into two groups according to the position of the retained testis (in the inguinal canal or in abdomen). Each cryptorchid was at the same time a case study and its own internal control because the non-retained contralateral scrotal testicle was used for comparing the expression of each gene/protein. For each receptor/enzyme under investigation, the gene expression by Real-Time PCR and the protein expression and localization by Western blotting and immunohistochemistry were analyzed and compared between the retained and the contralateral scrotal testis to highlight the effect of cryptorchidism on their expression. The blood of non-cryptorchid equines (controls) was used for comparing the serum concentration of calcium and VD between cryptorchid and non-cryptorchid subjects.

### 2.2. Reagents

All reagents were purchased from Sigma-Aldrich (Milano, Italy) unless otherwise stated.

### 2.3. Animals

Thirteen unilateral cryptorchid equines (age 2–8 yrs, breeds: quarter horse, Italian saddle, trotter, and murgese) were used for this study (Table 1).

Eight of them showed the testis retained in abdomen while the others had an inguinal retention. Inclusion factor for the enrollment was the eligibility for surgery. All subjects required castration as clinical intervention, so consent for the surgical procedure was requested of the equine owners and no license from the Bioethics Commission of the Department was necessary; nevertheless, written consent for the surgical procedure was released from each owner. In addition, the testicles of five normal equines obtained from the local abattoir were used as controls, along with corresponding blood-serum samples to assess the concentration of VD. Blood samples were taken prior to slaughter. The surgical procedure for castration was described in a previous paper [28] employing a flank laparoscopic procedure or a mini laparotomy as needed. In three of the equines with abdominal retention, testes were removed via a paramedian mini laparotomy under general anesthesia. The inguinal testes were removed via a scrotal approach, under general anesthesia. After removal, testes were immediately processed.

Small fragments of testicular tissue from the undescended testicle (Figure 1) and from the contralateral scrotal testicle were frozen in liquid nitrogen until use. The remaining tissue was fixed in 4% (*w*/*v*) phosphate-buffered (PBS) paraformaldehyde and processed for immunohistochemistry. The same sampling procedure was applied to normal testicles.

Before surgery, a blood sample was collected from the jugular vein of each subject; as in the control group to determine calcium and VD concentrations.

### 2.4. Calcium and VD Blood Determination

After recovering, blood samples were centrifuged at 1650× *g* to obtain serum that was kept frozen until use. Calcium concentrations were determined by a spectrocolorimetric assay (calcium ocpc, Assel s.r.l, Rome, Italy; code asr01050) and results visualized in Figure 2.

Vitamin-D determination was performed by a validated LC-MS/MS method [29]. Briefly, 100 μL of serum was precipitated with 150 μL acetonitrile containing the internal standard, d3-25-hydroxyvitamin D_3_ (d325D3). Then 25OHD_3_, 25OHD_2_, and d325D_3_ were extracted with 1.0 mL of hexane. Chromatographic separation was performed on a C18 analytical column with isocratic elution at a flow rate of 0.1 mL/min, utilizing a mobile phase consisting of 85% aqueous methanol/ammonium acetate/formic-acid buffer. Positive-ion electrospray ionization at 25 KV and selected reaction monitoring were used to follow the predominant transitions: collision energy (CE) = 10 at *m*/*z* 401→383 for 25OHD_3_, CE = 10 at *m*/*z* 413→395 for 25OHD_2_, and 404→386 for d325D3. The method was validated according to FDA guidance requirements. Calibration curves were constructed using six points. Accuracy and precision (within-run and between-runs) were all within 7.5%. Extraction recoveries averaged 57–64% for 25OHD_3_, 69–73% for 25OHD_2_, 55–56% for d325D_3_; linearity range for both 25OHD_3_ and 25OHD_2_ was 1.2–120.0 ng/ml, R^2^ > 0.99; results are visualized in Figure 3.

### 2.5. Real-Time PCR

Total RNA from testis tissue was extracted using Quick RNA MiniPrep Plus kit (code R1057, ZymoReserch, Irvine, CA, USA). RNA quality and concentration were evaluated using a spectrophotometer (DU640 Beckman, Indianapolis, IN, USA) and 1% agarose gel electrophoresis. Only RNA samples with A260/A280 ratios between 1.8 and 2.2, showing also clear ribosomal RNA bands, were used for further analyses. First-strand cDNA was synthesized from 1 μg of total RNA by the SensiFAST cDNA Synthesis kit (code BIO-65053, Meridian Bioscience, Memphis, TN, USA) following the manufacturer’s suggestions. For the minus-RT control, water was added instead of cDNA synthesis mix. Quantitative Real-Time PCR was performed with the StepOne thermal cycler (Applied Biosystem, Milan, Italy). In Table 2, for each gene, the PCR couple of primers and the expected amplicon size were displayed. Samples were amplified in duplicates on a Microamp fast optical 48-well reaction plate (Applied Biosystem, Milan, Italy).

Each 20 μL reaction contained: 10 µL KiCqStart^TM^SYBR Green qPCR Ready Mix with ROX (code KCQS02, Sigma-Aldrich, Milan, Italy), 1 µL (250 nM each Primer), 2 µL (100 ng) cDNA, 7 µL RNase free water. Cycling parameters were: 10 min at 95 °C followed by 50 cycles of 15 s at 95 °C and 1 min at 60 °C. Melting curve analysis was performed to verify primer specificity and the absence of primer dimers. Moreover, an aliquot of the amplified products was also electrophorezed onto a 1.5% agarose gel for visualization and molecular-weight confirmation (Figure 4). Amplification threshold cycle data were recovered from the StepOne Software v2.1. The GAPDH gene was used as reference gene for normalization. For each gene, the relative expression level was calculated by the 2^−ΔΔCt^ method, where Ct = threshold cycle; ΔCt = Ct_Mean (VDR or CYP2R1 or CYP27A1 or CYP27B1) − Ct_Mean (GAPDH); ΔΔCt = ΔCt test (abdominal or inguinal)-ΔΔCt_scrotal; so that the gene expression level = 2^−ΔΔCt^. Expression values are presented as fold changes relative to the scrotal sample.

### 2.6. Western Blotting

Fragments of testicular tissue were pulverized in liquid nitrogen and completely homogenized in cold lysis buffer 1x Phosphate buffer saline (PBS), 0.1% Triton-X, 50 µL/mL of the complete protease inhibitor cocktail (Santa Cruz Biotechnology, Inc., Dallas, TX, USA) as previously described [30]. Proteins were extracted from the lysed tissue by a 30 min centrifugation at 10,000× *g* and recovered in the supernatant. Protein concentration was determined by the Bradford method (Protein Assay, Bio Rad, Milano, Italy) measuring the absorbance of the solution by means of DU600 spectrophotometer (Beckman Coulter, Milano, Italy). Forty micrograms of proteins from each subject were loaded into a precast 4–20% polyacrylamide gel (Criterion, Bio-Rad, Milano, Italy), electrophorezed under constant voltage and transferred to a PVDF membrane (Millipore, Milano, Italy) by the Trans-Blot^®^SD Semi-Dry Transfer cell (Bio-Rad, Milano, Italy).

For each primary antibody, the membrane was hybridized with a blocking buffer solution (0.1% Tween-20 in PBS) containing 1 µg (dilution 1:400) of the corresponding antibody. To quantify VDR, CYP2R1, CYP27A1, and CYP27B1 proteins, the following antibodies were used separately and hybridized to the membrane by the Snap i.d. system (Merck-Millipore, Darmstadt, Germany): (1) a mouse monoclonal antibody raised against amino acids 344–424 of VDR of human origin (ARG63832, Arigo Biolaboratories, Zhubei City, Taiwan), (2) a rabbit polyclonal antibody raised against amino acids 1–100 of CYP2R1 of human origin (sc-98919, Santa Cruz Biotechnology Inc., Dallas, TX, USA), (3) a rabbit polyclonal antibody raised against amino acids 335–451 mapping within an internal region of CYP27A1 of human origin (sc-292976, Santa Cruz Biotechnology Inc., Dallas, TX, USA), (4) a rabbit polyclonal antibody raised against a linear peptide corresponding to human CYP27B1 (ABN182, Merck-Millipore, Darmstadt, Germany). To reveal the immunocomplex, the Vectastain Elite ABC kit was used (Vector Laboratories Inc., Burlingame, CA, USA). For signal normalization and loading controls, membranes were also hybridized to anti GAPDH antibody diluted 1:1500 (sc-47724, Santa Cruz Biotechnology Inc., Dallas, TX, USA). A negative control was also performed omitting the primary antibody.

Because the employed antibodies were not specific for the equine species, but raised against proteins of human origin, we checked a theoretical identity between the equine and human protein sequences and aligned them by the BLAST software v2.16 from NCBI. The use of the human CYP27A1 antibody in equines was recommended by the company, nevertheless we found 72.46% identity between the human and equine CYP27A1 sequences (sequence accession number: NP_000775.1-human and XP_023498116.2-equine). For the other antibodies used in this study, we found 90.63% identity between the human and equine VDR sequences (sequence accession number: NP_000367.1-human and NP_001157431.1-equine); 95.37% identity between the human and equine CYP2R1 sequences (sequence accession number: NP_078790.2-human and XP_023501851.2-equine); and 89.17% identity between the human and equine CYP27B1 sequences (sequence accession number: NP_000776.1-human and NP_001157429.2-equine). The optical density of each protein band was quantified using Quantity one software v4.0 (BioRad, Milano, Italy). The values are presented in arbitrary units as the ratio of the specific protein to the corresponding GAPDH optical density and visualized by box-and-whisker plots.

### 2.7. Histology

#### 2.7.1. Tissue Preparation

Tissue fragments (1 cm thick) were obtained from the abdominal, inguinal, and scrotal testes of cryptorchid and normal equines and fixed in 4% (*w*/*v*) phosphate-buffered (PBS) paraformaldehyde at room temperature (RT) for 24 h. Then the specimens were dehydrated in an ascending ethanol series, cleared in xylene, and embedded in paraffin wax. Serial sections (5 μm thick) were cut and, after de-waxing with xylene and hydration in an ethanol series of descending concentrations, were stained with hematoxylin–eosin for morphological analysis and by immunohistochemistry to localize the presence of VDR- and VD-activating (CYP2R1, CYP27A1, CYP27B1) enzymes.

#### 2.7.2. Immunolocalization of VDR, CYP2R1, CYP27A1, and CYP27B1

De-waxed and re-hydrated tissue sections were incubated for 20 min in 3% H_2_O_2_ to inhibit endogenous peroxidase activity. After washing in PBS, non-specific binding sites were blocked by incubation in normal horse serum (NHS) (Vector Laboratories, Burlingame, CA, USA) for 30 min at RT. Sections were incubated overnight at 4 °C in a moist chamber with anti-VDR, CYP2R1, CYP27A1, and CYP27B1 primary antibodies, the same as those used for the Western blotting, diluted 1:200 in PBS containing 2.5% NHS. After a washing step with PBS for 15 min, the biotinylated universal antibody (Vector-Laboratories, Burlingame, CA, USA) was added for 30 min at RT, followed, after a new 15-min PBS wash, by 30 min of incubation with Vectastain Elite ABC Reagent (Vector-Laboratories, Burlingame, CA, USA). After washing in PBS, staining was visualized by incubating the sections in DAB solution (Vector-Laboratories, Burlingame, CA, USA). Then, the sections were dehydrated and mounted. The negative control was performed by replacing the primary antibodies with NHS in PBS. Under these conditions, staining was abolished.

### 2.8. Statistical Analysis

Data were analyzed by the Statistical Package for Social Sciences (SPSS, version 22) software. Because the study concerns a small equine sample size, data were analyzed by non-parametric methods. The Mann–Whitney test was employed to evaluate statistically significant differences between independent groups. Data were expressed by box-and-whisker plots to highlight median and IQR values. Statistical significance was settled at *p* values ≤ 0.05.

## 3. Results

### 3.1. Calcium and VD Blood Determinations

The values of serum calcium concentration recorded in equines are shown in Figure 2; they are expressed as mg/dL. In the control group of non-cryptorchid equines, the values of median and IQR were 12.1; 1.5 respectively, while in unilateral cryptorchid equines with inguinal retention the values were 10; 2.87 and in the equines with abdominal retention the median and IQR values were 11.9; 2.05. The Mann–Whitney test for unpaired groups showed that the comparison of calcium concentrations between the two groups of equines with the different testis retention did not show a statistically significant difference (*p* = 0.105); as well as the comparison between the group of equines with abdominal retention and the control group (*p* = 0.340). In contrast, a statistically significant difference (*p* = 0.028) was found when comparing the group of equines with inguinal retention to the control group.

Serum VD concentration measured in unilateral cryptorchid equines and in the control group are expressed in ng/mL and shown in Figure 3 by box-and-whisker plots. Median and IQR values obtained in equines with the testis retained in the inguinal canal were 5.66; 2.8 respectively, while values found in the group of equines with the testis retained in abdomen were 7.38; 3.27. The values of the control group were 5.7; 0.8. The Mann–Whitney test showed that all comparisons between groups were not statistically significant (inguinal vs. abdominal *p* = 0.464; inguinal vs. control *p* = 0.917; abdominal vs. control *p* = 0.464).

### 3.2. Real-Time PCR

The analysis by Real-Time PCR demonstrated that the equine testis is a site of transcription for VDR, CYP2R1, CYP27A1, and CYP27B1 genes. Figure 4 shows that all genes were transcribed and for each gene the corresponding amplicon was of the expected size as reported in Table 1.

Moreover, as reported in Figure 5 for the *VDR* gene, the expression level of the testicle retained in the inguinal canal even higher than that of the contralateral scrotal testicle, resulted not statistically significant different because of the high standard deviation values (inguinal-retained vs. scrotal: 3.60; 5.65 vs. 1; 0 *p* = 0.487; abdominal-retained vs. scrotal: 1.34; 0.32 vs. 1; 0 *p* = 0.487; inguinal-retained vs. abdominal-retained: 3.60; 5.65 vs. 1.34; 0.32 *p* = 0.513).

The expression of the *CYP2R1* gene was not related to the position of the testicle (inguinal-retained vs. scrotal: 0.54; 0.61 vs. 1; 0 *p* = 0.487; inguinal-retained vs. abdominal-retained: 0.54; 0.61 vs. 0.96; 0.49; *p* = 0.827; abdominal-retained vs. scrotal: 0.96; 0.49 vs. 1; 0 *p* = 0.487).

The Mann–Whitney test evidenced for *CYP27A1* gene a statistically significant decrease in the level of expression when the undescended testicle was compared to the contralateral scrotal testis (inguinal-retained vs. scrotal: 0.08; 0.11 vs. 1; 0 *p* = 0.037; abdominal-retained vs. scrotal: 0.001; 0.34 vs. 1; 0 *p* = 0.037), while no statistically significant difference was observed in the comparison between the two different positions of testicle retention (inguinal-retained vs. abdominal-retained: 0.08; 0.11 vs. 0.001; 0.34; *p* = 0.513).

As for the *CYP27B1* gene, even if the expression average value of the inguinal-retained testicle was higher than that of the contralateral scrotal testis, the comparison was not statistically significant because of the high standard deviation value (inguinal-retained vs. scrotal: 3.06; 3.68 vs. 1; 0 *p* = 0.487), on the contrary the expression level of the testis retained in abdomen was lower than that of the contralateral scrotal testicle (abdominal-retained vs. scrotal: 0.28; 0.20 vs. 1; 0 *p* = 0.037). The gene expression level was also statistically different between testes retained in the different positions (inguinal-retained vs. abdominal-retained: 3.06; 3.68 vs. 0.28; 0.20; *p* = 0.05).

### 3.3. Western Blotting

The hybridization of proteins from equine testes with the antibodies against the VDR- and VD-metabolizing enzymes showed the positive signals of the expected molecular size bands corresponding to 60 kDa (VDR), 55 kDa (CYP2R1), 57 kDa (CYP27A1), and 58 kDa (CYP27B1) (Figure 6).

The data of the densitometric analysis were shown in the box-and-whisker plots (Figure 7) that report also the results of the statistical analysis.

Panel A shows the recorded expression values of VDR protein. The median and IQR values of the inguinal-retained testicles were 0.63 and 0.22, those related to the contralateral scrotal testicle were 0.72 and 0.22, and *p* = 0.530; the values related to the abdominal-retained testicle were 0.62 and 0.10 and of the contralateral scrotal testicle 0.69 and 0.16, and *p* = 0.036. The pooled values of the retained testicles were 0.63 and 0.14, those of the scrotal testicles 0.70 and 0.075 and *p* = 0.091.

As for the CYP2R1 protein (panel B), the median and IQR values of the inguinal-retained testicles were 0.60 and 0.28, those related to the contralateral scrotal testicle were 0.55 and 0.35, and *p* = 0.827; the values related to the abdominal-retained testicle were 0.46 and 0.14 and of the contralateral scrotal testicle 0.56 and 0.30, and *p* = 0.206. The comparison between the two retained testicles was not statistically significant (inguinal vs. abdominal *p* = 0.102). The pooled values of the retained testicles were 0.48 and 0.21, those of the scrotal testicles 0.55 and 0.37, and *p* = 0.758.

For the CYP27A1 protein (panel C) the median and IQR values corresponding to the inguinal-retained testicles were 1.22 and 0.023 respectively while those of the contralateral scrotal testicle were 1.52; 0.199. Comparing the protein expression in the two differently located testicles, a statistically significant difference (*p* = 0.009) was found. As for abdominal retention, the expression values were 1.36 and 0.079 while those of the contralateral testicle were 1.55 and 0.13 and this comparison (abdominal vs. scrotal retention) was statistically significant (*p* = 0.002). The comparison between the expression of CYP27A1 in the two retained testicles was statistically significant different (inguinal vs. abdominal *p* = 0.003). Pooling together the data of the retained testicles the median and IQR values were 1.28 and 0.11 while those of the scrotal testicles were 1.54 and 0.11 and the difference was statistically significant (*p* = 0.000).

As for the CYP27B1 protein (panel D) the median and IQR values of the inguinal-retained testicles were 1.22 and 0.023, those related to the contralateral scrotal testicle were 0.74 and 0.03, and *p* = 0.05 showing a statistically significance at the minimum limit value; those of the abdominal-retained testicle were 0.6 and 0.096 and of the contralateral scrotal testicle 0.70 and 0.20, and *p* = 0.275. The comparison between the expression of CYP27B1 in the two retained testicles was statistically significantly different at the minimum limit value (inguinal vs. abdominal *p* = 0.05). The pooled values of the retained testicles were 0.59 and 0.07, those of the scrotal testicles 0.74 and 0.15, and *p* = 0.109. Results are summarized in Table 3.

### 3.4. Histology

The histological analysis of the retained and contralateral testes did not show evidence of testicular neoplasia (Figure 8), although the seminiferous tubules of both the abdominal- and inguinal-retained testes displayed altered morphology compared to the contralateral scrotal testes (Figure 8A,B). Specifically, the seminiferous epithelium of all retained testes was vacuolized in appearance and was constituted of Sertoli cells and spermatogonia (Figure 8A,B). The absence of the other spermatogenic cell types (spermatocytes and spermatids) is clearly indicative of the spermatogenesis block. On the contrary, as in the normal testes, the germinal epithelium of the contralateral scrotal testes was normal in aspect and displayed germ cells in various stages of maturation, including spermatozoa (Figure 8C,D).

The immunolocalization pattern of the VDR, CYP2R1, CYP27A1, and CYP27B1 is summarized in Table 4 and is shown in Figure 9, Figure 10, Figure 11 and Figure 12.

VDR (Figure 9) was detected in the interstitial cell of all investigated testes, with the highest immunostaining in the abdominal and contralateral testis of cryptorchid equines; in the Sertoli cells, mainly in the retained testes; in the spermatocytes of all scrotal testes, although it was less evident in seminiferous epithelium of normal (control) stallions; strong anti-VDR immunostaining was observed in the spermatids of all scrotal testes.

The immunostaining of CYP2R1 revealed a high presence of this enzyme in the interstitial cells of all investigated testes, a faint visible immunoreaction in the Sertoli cells of cryptorchid testes, in the spermatogonia of cryptorchid and scrotal testes and in the spermatocytes of the scrotal testes. A more distinct immunostaining was observed in the round spermatids of all scrotal testes (Figure 10).

CYP27A1 was strongly expressed in the interstitial cells of all cryptorchid and scrotal testes. Regarding its presence in the seminiferous epithelium, a faint immunoreaction was detected in the Sertoli cells of analyzed testes, in the spermatogonia of abdominal-retained testes and the control testes, and in the spermatocytes of all scrotal testes. A well-visible immunostaining was observed in the round spermatids of normal testes (Figure 11).

CYP27B1 immunostaining evidenced a staining intensity in the interstitial cells similar to CYP27A1. In the seminiferous epithelium, CYP27B1 was highly expressed in the Sertoli cell of abdominal testes and faintly in the Sertoli cells of other testes and the spermatocytes of scrotal testes from inguinal cryptorchid equines. The spermatocytes of normal equines displayed the strongest CYP27B1 immunoreaction. A moderate intensity of the immunostaining was observed in the round spermatids of the contralateral cryptorchid testes and in all types of spermatids of control testes (Figure 12).

## 4. Discussion

The aim of this work was to investigate on the presence and localization of VDR and VD bioactivating enzymes in the equine testis of normal and cryptorchid conditions. Furthermore, evaluations were made in unilateral cryptorchid equines, comparing the retained to the contralateral scrotal testicle, with the aim of establishing, for each subject, its own internal control.

Cryptorchidism, which has a polygenic nature, has been recognized as a risk factor for infertility and developing testicular germ-cell tumors; in fact, men with a history of cryptorchidism account for 10% of patients affected by testicular germ-cell tumors [31]. Among animals, the risk of developing testicular cancer is 9.2 to 13.6 higher in cryptorchids than in normal dogs (see [32] for references) and in cryptorchid equines, teratoma, seminoma, and Sertoli cell tumor were also found [33].

VD also has non-calciotropic actions [34]; therefore, our focus on VDR- and VD-activating enzymes is related to both the requirement of these molecules for the proper physiology of the testis and on the open question on VD as antiproliferative molecule [34]. Several studies in vitro and in vivo showed that male reproductive functions relay on VD, because it affects testosterone synthesis, spermatogenesis, and the maturation of spermatozoa [21]. In rats the positive association between lower circulating testosterone levels and hypovitaminosis D has been demonstrated since 1986 [35], while in chickens, although circulating levels of testosterone were similar among vitamin-D-depleted and vitamin-D-replaced subjects, calcium homeostasis was deeply affected because of the reduced expression of the calcium-binding protein calbindin-D_28k_ [36].

The biology of VD cannot be extrapolated from humans to equines because it is well known that total VD content in equines is lower, and it would be considered a deficiency in humans. On the other hand, VD produced by the skin is minimal compared to other animal species so that the equine VD status depends on dietary VD_2_ or supplemental VD; for this reason, the management factors become important determinants in equines. In this study we evaluated both serum VD and calcium concentrations to establish whether cryptorchidism could impact these parameters. We found that there was no difference between cryptorchids and normal equines in VD concentrations while cryptorchid subjects with the inguinal-retained testicle revealed a lower serum calcium concentration compared to normal subjects. The meaning of this result needs more investigation to establish whether it could be a consequence of a higher calcium request from the seminal plasma of cryptorchid subjects to ensure calcium-related testicular functions that are compromised by this pathology. Furthermore, as discussed in one of our previous studies [28], the different location of the testicle when it is retained exposes it to different compressive forces, which could account for the slight differences observed between inguinal and abdominal retention. On the other hand, in humans it is well known that calcium seminal plasma is two-to-three-fold higher compared to serum [5].

Moreover, although the effect of VD on equine calcium and phosphorus metabolism appears limited, its effect on other physiological non-skeletal processes is unknown included to the modulation of testicular function, for which the testicular expression of VDR is essential.

Whether equine tissues contribute similarly to other species to VD bioactivation is a question that was already addressed in 1998 [20]; the authors evidenced, in an in vitro model, lack of 25OHDx-1-a-hydroxylase (namely CYP27B1) activity in the kidney cortex; on the contrary, several years later, it was demonstrated that equine kidney expresses CYP27B1 and CYP24A1 mRNA [37] suggesting that despite low serum VD concentration, it plays a significant role in equine calcium metabolism.

In equine species, VD contribution to the maintenance of circulating calcium and phosphorus levels does not seem high; therefore, it is likely interesting to investigate the other VD functions—beyond their well-studied role in calcium homeostasis and bone metabolism—as a possible paracrine and autocrine actors in the testis, as it also happens in humans [38]. More in detail, it can be investigated whether VDR is expressed similarly in equine testis as in humans and whether in cryptorchid subjects, a compensatory mechanism (such as upregulation of VDR- and/or VD-activating genes) exists. It could act to safeguard fertility and to prevent the neoplastic progression of the retained testis considering that in humans the hormone can modulate the expression of up to 10% of the genome comprising anti-cancer genes [38].

To our knowledge this is the first investigation describing and comparing the expression of VDR- and VD-activating enzymes (CYP2R1, CYP27A1, CYP27B1) in retained and scrotal testis of unilateral cryptorchid equines both at gene and protein levels while also evaluating the cellular localization of the proteins.

The results demonstrated that equine testes express the genes coding for VDR- and for VD-activating enzymes (Figure 4) and that cryptorchidism affects mRNA levels of the investigated genes. The amount of VDR mRNA was not correlated to the localization of the retained testicle. The corresponding protein was also expressed in the equine testis with a reduced level in the abdominal-retained testicle compared to its contralateral scrotal testicle, while the expression in the inguinal-retained testicle was like the contralateral scrotal one.

These results obtained in equines reinforce the idea that VDR is expressed in “virtually every tissue of the body” as in humans [38], and that the presence of the receptor in the retained testicles could allow its hormone ligand to safeguard the testicle from the possible neoplastic progression. In support of this, we did not find neoplastic cells in all samples.

The gene coding for the VD-activating enzyme CYP2R1 was also expressed in the testicle at comparable levels in the scrotal and in both retained testicles. The corresponding protein was also expressed in both retained testicles at comparable levels.

As for CYP27A1, the expression was reduced in the retained testis compared to the contralateral testicle, despite the site of retention. The expression of this gene was also demonstrated in equine testes of normal and castrated subjects at different ages [25]. Concerning the corresponding protein, we found a positive hybridization signal of expected molecular size and a decreased level of expression when each retained testicle was compared to the contralateral scrotal testis. Overall, in the retained testicles, the CYP27A1 protein was significantly under-expressed.

Comparing the expression of CYP27A1 and CYP2R1 (each one normalized to its own scrotal testicle), we found that in the inguinal-retained testicle the average expression value of CYP2R1 was higher than that of CYP27A1, as also demonstrated in human testes [21]; however, due to the high standard deviation observed we were unable to demonstrate that also in the equines the difference was statistically significantly different. In any case, despite a lower expression recorded in the retained testicles, the combined presence of both proteins led us to suppose that it may safeguard the testis from an abnormal cellular proliferation.

CYP27B1 was also expressed in equine testes as demonstrated by experiments of gene expression and Western blotting and immunohistochemistry. As well for CYP2R1 and VDR, the results do not match the findings reported by Rouge et al. [25] who halted the study at the gene level because they found no evidence of expression; however, in the study, they did not include a positive control to verify the efficiency of the employed primers. Our results evidenced that CYP27B1 was more expressed in the abdominal-retained testis compared to the inguinal one. The higher expression in the abdominal-retained testicle could be explained by the higher request to complete VD bioactivation considering that the abdomen is the worst testicular localization for spermatogenesis because of the higher temperature.

Taken together, these results demonstrated that the cryptorchid condition that compromises spermatogenesis, the metabolic state of the testis with an upregulation of some mitochondrial gene for ATP production [39], and a general change in gene expression, with some genes upregulated and others downregulated [40], lowers but does not abolish the expression of VD-related proteins. This helps to support the idea that, also in equines, the testis is an extra site of VD bioactivation necessary for VD function beyond calcium homeostasis and bone metabolism.

Except for the present study on equines, the contemporary localization of testicular VDR- and VD-activating (CYP2R1, CYP27A1, CYP27B1) enzymes has been investigated only in men [21]. The few published studies on this issue report the VDR immunolocalization in mice [41,42], rats [41,43], and roosters [44]. The immunolocalization of CYP2R1 was also detected in human testicles by Foresta et al. [22], and the testicular localization of CYP27A1 and CYP27B1 has not been reported in other species.

Leydig cells are the testicular cell type in which VDR- and VD-activating enzymes were strongly expressed in all the analyzed testes, without apparent differences in staining intensity among cryptorchid, contralateral scrotal, and control testes. In a previous study the expression of CYP27A1 has been demonstrated in the interstitial tissue of horses [25]. In an immunohistochemical study on the human testis, the presence of VDR- and VD-metabolizing enzymes has been detected in Leydig cells [21]. This suggests that VD plays a direct role in regulating steroidogenesis. In men with testicular problems, lower systemic VD levels have been observed than in healthy controls due to reduced expression of CYP2R1 in Leydig cells [22]. This indicates that the Leydig cell could play a major role in the healthy testicle, since progressive decrease of CYP2R1 expression in relation to the severity of testiculopathy was found [22]. More recently, it has been hypothesized that Leydig cells might drive local production of 25-hydroxy-vitamin D in the testis [45].

The VDR is not present in the Leydig cells of all mammals, since in two different studies its absence was observed in Leydig cells of rats [41,43]. Johnson et al. [43] explained this finding by suggesting that VD indirectly regulates testosterone synthesis, since changes in plasma concentrations of luteinizing hormone (LH), which stimulates testosterone synthesis by Leydig cells, run parallel to those of testosterone in response to changes in VD levels [35].

Regarding the distribution of VDR- and VD-activating enzymes in the seminiferous epithelium, immunohistochemistry revealed heterogeneous expression of the studied epitopes across different cell types and among the animals from which the testes were taken.

The Sertoli cells displayed the highest presence of VDR in the retained testes; this receptor was barely visible in the Sertoli cells of all scrotal testicles. VDR has not been found in the human Sertoli cells [21], whereas anti-VDR immunostained Sertoli cells have been detected in mice [41,42] and rats [41,43]. Interestingly, in an experimentally induced cryptorchidism, the Sertoli cells of mice displayed a significantly enhanced relative expression of VDR mRNA compared to normal testes [42]. The authors speculated that VD may have an environmental protection of germ cells by influencing Sertoli cells rather than stimulating germ-cell proliferation. It has been reported that Sertoli cells are important targets of VD in the testis [46,47,48] and that VD acts on Sertoli cells by upregulating Abca1 gene expression [42]. Regarding the VD-activating enzymes, they have not been found in the Sertoli cells of men [21,22], while we found traces of CYP2R1, CYP27A1, and CYP27B1, albeit generally in small amounts, in the Sertoli cells of all investigated equine testes. It is difficult to explain this discrepancy, although it cannot be ruled out that it may be caused by a species-specific characteristic, together with the antibodies and methods used.

The spermatogonia expressed VDR and CYP2R1 in all analyzed testes, with a higher level of VDR in cryptorchid and contralateral scrotal testes compared with the testes of normal equines, and faintly visible presence of CYP2R1 in all types of testicles. Regarding CYP27A1 and CYP27B1 epitopes, they were not present in spermatogonia of cryptorchid and scrotal testicles. The simultaneous presence of VDR and CYP2R1 in spermatogonia and Sertoli cells suggests that VD may play a role in equine spermatogenesis. VDR has been detected in the spermatogonia of rodents [42,43] and roosters [44]. In addition to VDR, human spermatogonia also expresses CYP27B1 but not CYP2R1 or CYP27A1 [21]. These results suggest that the action of VD and its activating enzymes in the testis needs further clarification.

Spermatocytes expressed VDR and CYP2R1, CYP27A1, and CYP27B1 in the scrotal testis of unilateral cryptorchid equines and in the testis of normal stallions. Since VDR and CYP2R1 showed a similar staining intensity in spermatocytes and spermatogonia, it can be assumed that these epitopes are constitutive components of equine spermatogenetic cells. Compared to spermatogonia, spermatocytes contained additional VD-activating enzymes such as CYP27A1 and CYP27B1. The presence of these two enzymes was more evident in control equines then in scrotal testes of unilateral cryptorchids. This finding may be related to a high fertility of control then unilateral cryptorchid equines. VDR has been detected in spermatocytes of rats [43] and roosters [44]. In human testes, spermatocytes have a high expression of CYP2R1 and few levels of CYP27A1 and CYP27B1 [21].

During spermiogenesis, all morphologically distinguishable types of spermatids (round and elongated spermatids) expressed VDR, CYP2R1, CYP27A1, and CYP27B1 in control equines. These findings evidence that an extrarenal activation of VD occurs in equines and that it may be important for spermatogenesis and sperm maturation. Our immunohistochemical results are consistent with those reported in men, whose round and elongated spermatids express VDR- and VD-activating enzymes [21].

It is interesting to note that, in the scrotal testes of animals with unilateral cryptorchidism, VDR, CYP2R1, and CYP27B1 were found only in round spermatids, whilst CYP27A1 was never detected. The comparison of these findings with those that we observed in the healthy control equines and by Blomberg Jensen et al. [21] in humans, suggests that the normal maturation of spermatids requires VDR- and VD-activating enzymes and that the spermiogenesis occurring in the scrotal testes of animals with unilateral cryptorchidism has an altered VD metabolism. In addition, the absence of the CYP27A1 may be a biomarker of defective molecular composition of equine spermatids. It is known that VD may be a regulator of the intracellular calcium content in mature sperm [49,50] and exerts a consistent beneficial effect on sperm quality in humans [51]. Further experimental studies are needed to clarify the functional role of CYP27A1, and the other molecules involved in VD metabolism in the equine spermiogenesis [21].

## 5. Conclusions

In conclusion, this study demonstrates, for the first time, that the VD metabolic system is present in the scrotal and cryptorchid testicles of equines. In particular, VDR and CYP2R1 are highly expressed in Leydig cells and, to a lesser extent, in all cell types comprising the seminiferous epithelium, whilst CYP27A1 and CYP27B1 are more abundant in spermatids from normal testes than in those from the scrotal testes of animals with unilateral cryptorchidism. Finally, no difference was observed in the cellular localization of the VD-metabolizing machinery between cryptorchid and scrotal testicles. These results provide insights for further studies, which are necessary to clarify the role of vitamin D in equine reproduction.

## Figures and Tables

**Figure 1:**
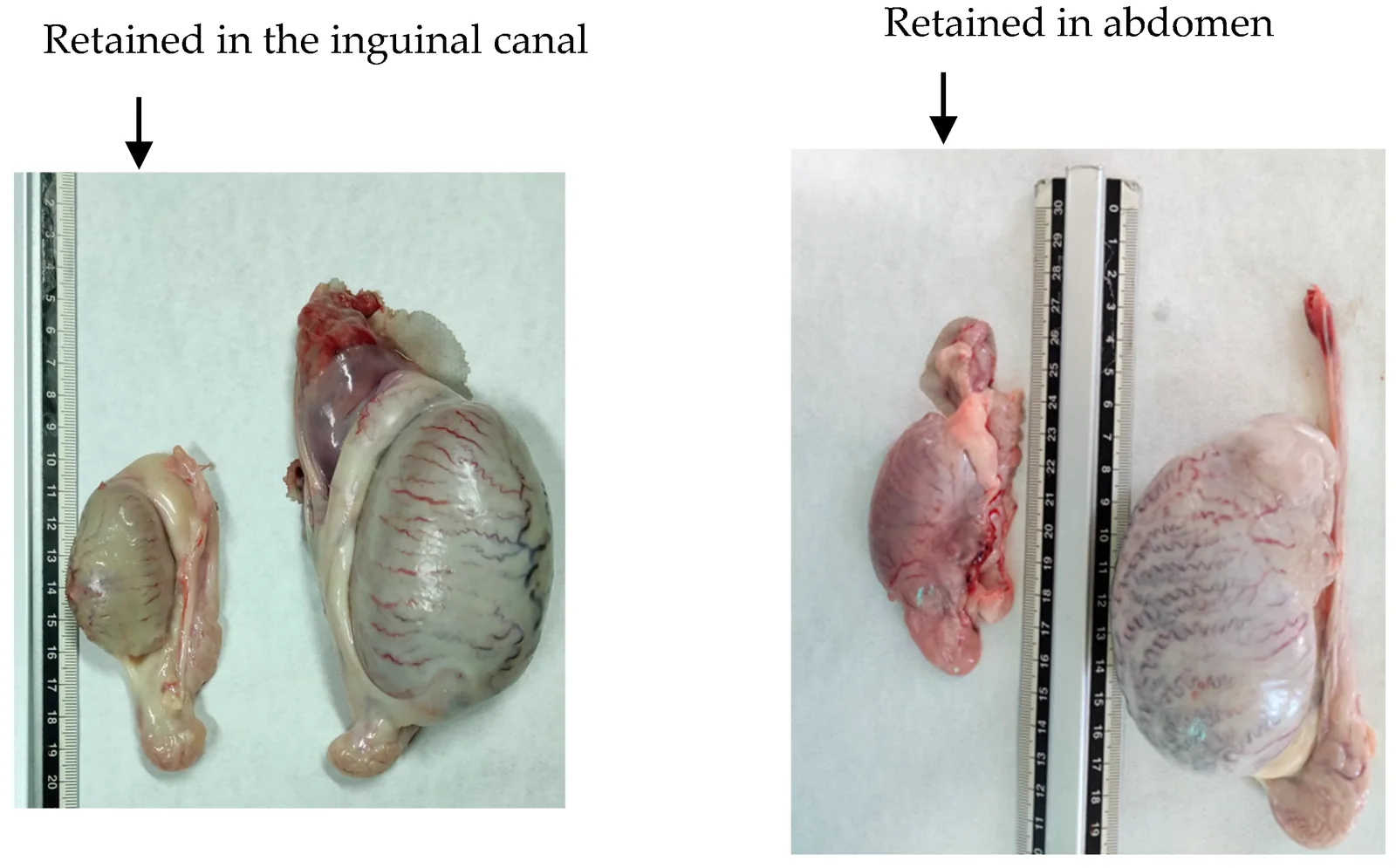
Both figures show testicles from unilateral cryptorchid equines. In the left panel the arrow indicates the testicle retained in the inguinal canal, the contralateral scrotal testicle is also shown; in the right panel the arrow indicates the testicle retained in abdomen. In both cases the contralateral scrotal testicle has normal size compared to the retained one.

**Figure 2:**
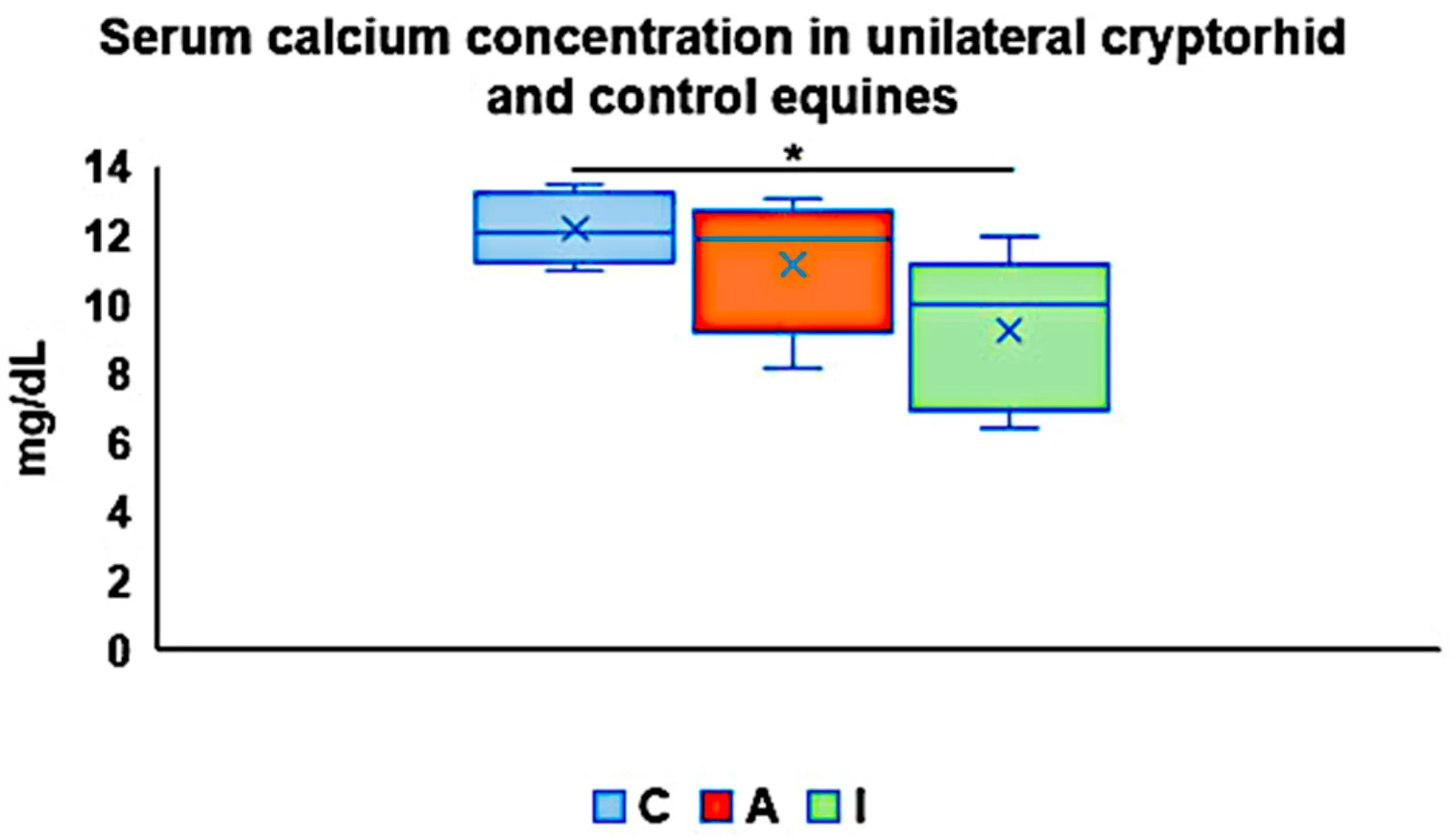
Calcium serum concentration in unilateral cryptorchid (n = 13) and control (n = 5) equines. In the box-and-whisker plots the concentration of calcium is reported in mg/dL * = *p* ≤ 0.05. C = control equines, I = unilateral cryptorchid equines with inguinal-retained testes, A = unilateral cryptorchid equines with abdominal-retained testes. In each box, X indicates the mean of the values.

**Figure 3:**
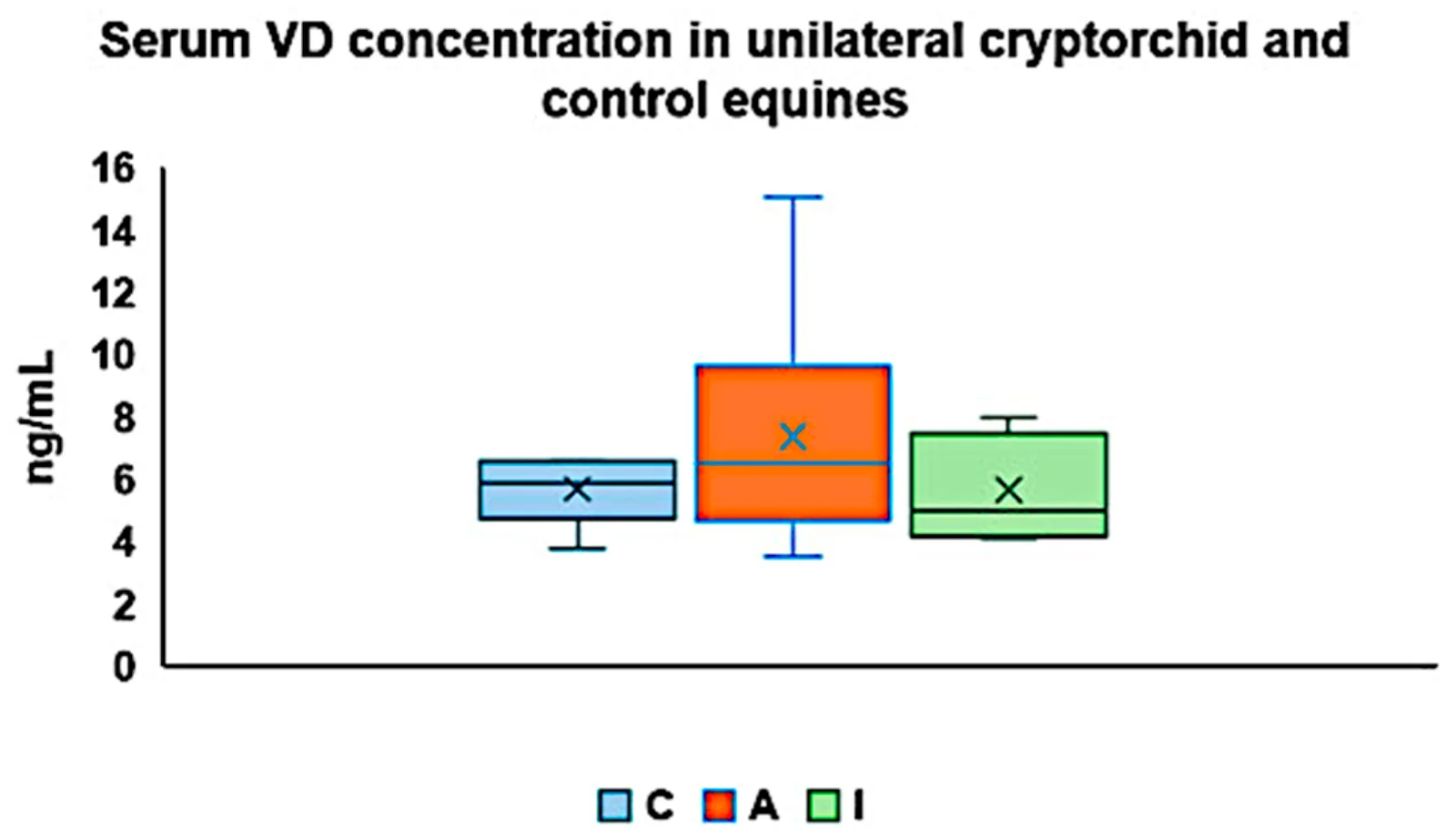
Serum concentration of VD in unilateral cryptorchid (n = 13) and control (n = 5) equines. In the box-and-whisker plots the concentration of serum VD is shown in ng/mL. C = control equines, I = unilateral cryptorchid equines with inguinal-retained testes, A = unilateral cryptorchid equines with abdominal-retained testes. In each box, X indicates the mean of the values.

**Figure 4:**
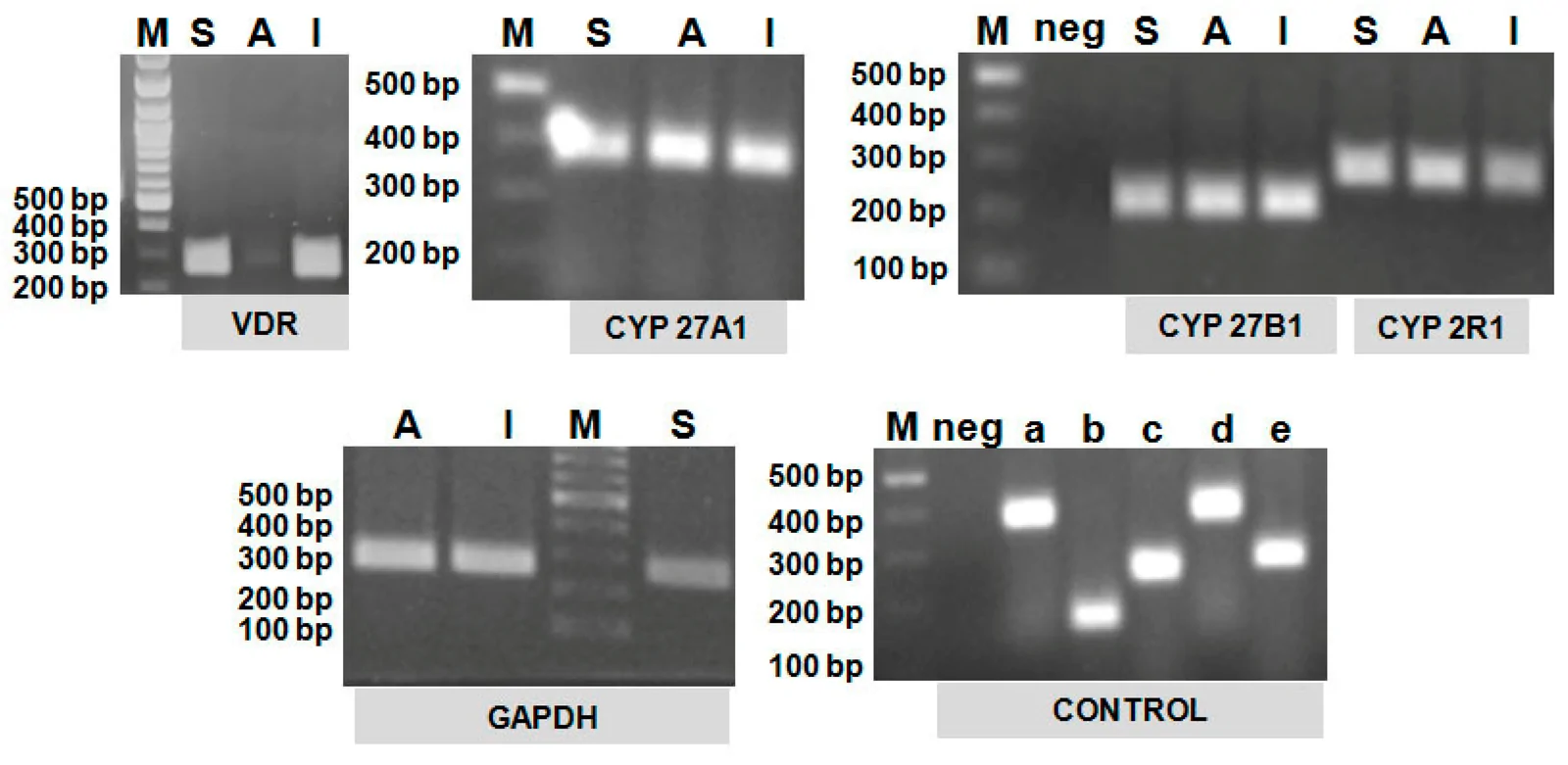
Agarose gel electrophoresis of Real-Time PCR products. In each lane of the gel, an aliquot of the Real-Time PCR product from one of the following genes: VDR (330 bp), CYP27A1 (325 bp), CYP27B1 (200 bp), CYP2R1 (245 bp), and GAPDH (254 bp) was loaded and electrophorezed under constant voltage. M = molecular-weight marker, neg = negative control, Control = testicle from control equines, S = scrotal testicle, A = abdominal-retained testicle, I = inguinal-retained testicle, a = CYP27A1, b = CYP27B1, c = CYP2R1, d = VDR, e = GAPDH.

**Figure 5:**
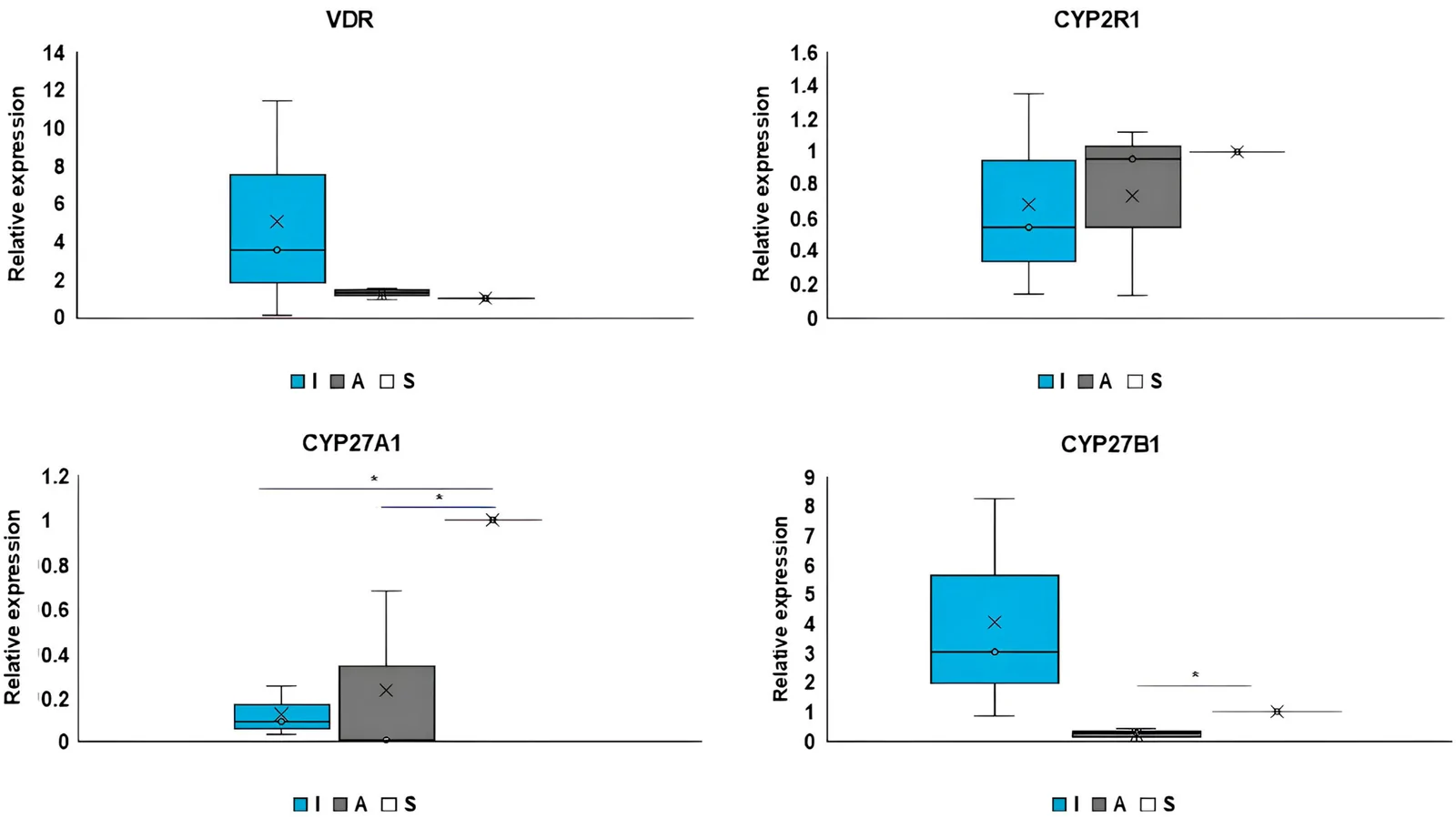
Relative gene expression of *VDR*, *CYP2R1*, *CYP27A1*, and *CYP27B1*, in equine testes. For each gene the box-and-whisker plots show the relative gene expression in undescended and scrotal testicles. * = *p* ≤ 0.05. S = scrotal testicle, I = inguinal-retained testicle, A = abdominal-retained testicle. In each box, X indicates the mean of the values.

**Figure 6:**
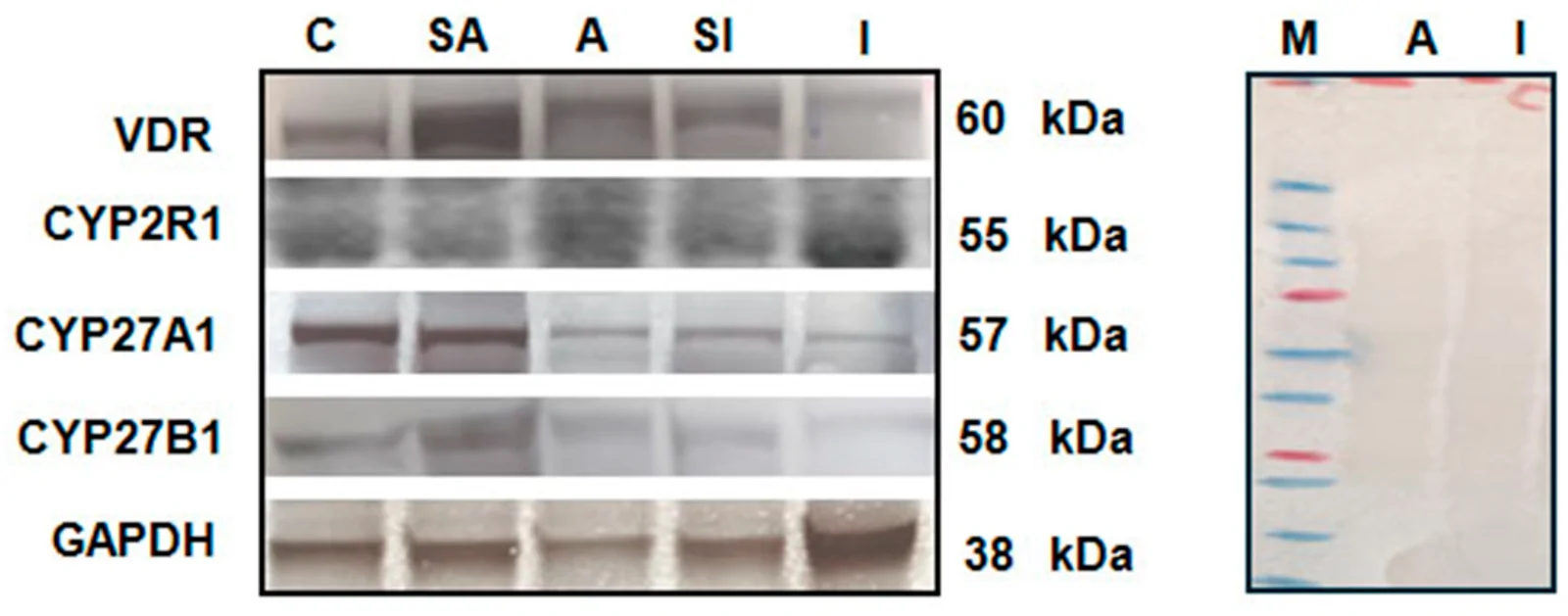
Representative Western blotting showing the expression of VDR- and VD-metabolizing enzymes in equine testes from unilateral cryptorchid subjects (left panel) and negative control where primary antibody was omitted (right panel). A = abdominal-retained testicle, I = inguinal-retained testicle, SA = contralateral scrotal testicle of the abdominal-retained testicle, SI = contralateral scrotal testicle of the inguinal-retained testicle, C = control testicle, M = dual color protein molecular-weight marker. Approximate molecular sizes are reported on the right side and expressed in kilodaltons.

**Figure 7:**
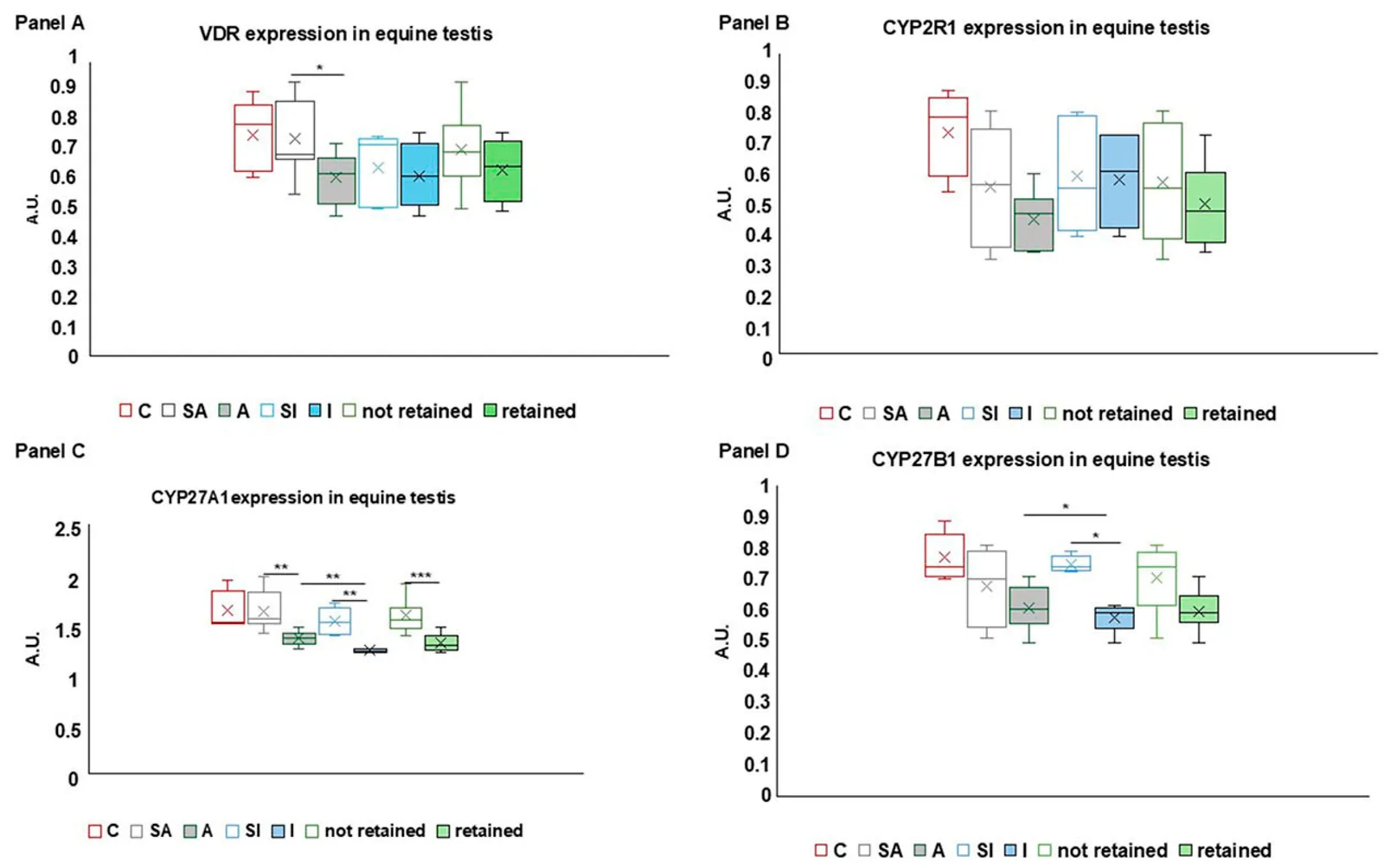
Representative box-and-whisker plots showing the densitometric analysis of VDR- and VD-metabolizing enzymes protein signals in equine testes from cryptorchid and control subjects. The expression of VDR (Panel (**A**)), CYP2R1 (Panel (**B**)), CYP27A1 (Panel (**C**)), and CYP27B1 (Panel (**D**)) are expressed in arbitrary units and shown together with level of statistical significance. * = *p* ≤ 0.05, ** *p* ≤ 0.01, *** *p* ≤ 0.001. A = abdominal-retained testicle, I = inguinal-retained testicle, SA = contralateral scrotal testicle of the abdominal-retained testicle, SI = contralateral scrotal testicle of the inguinal-retained testicle, C = control testicle. In each box, X indicates the mean of the values.

**Figure 8:**
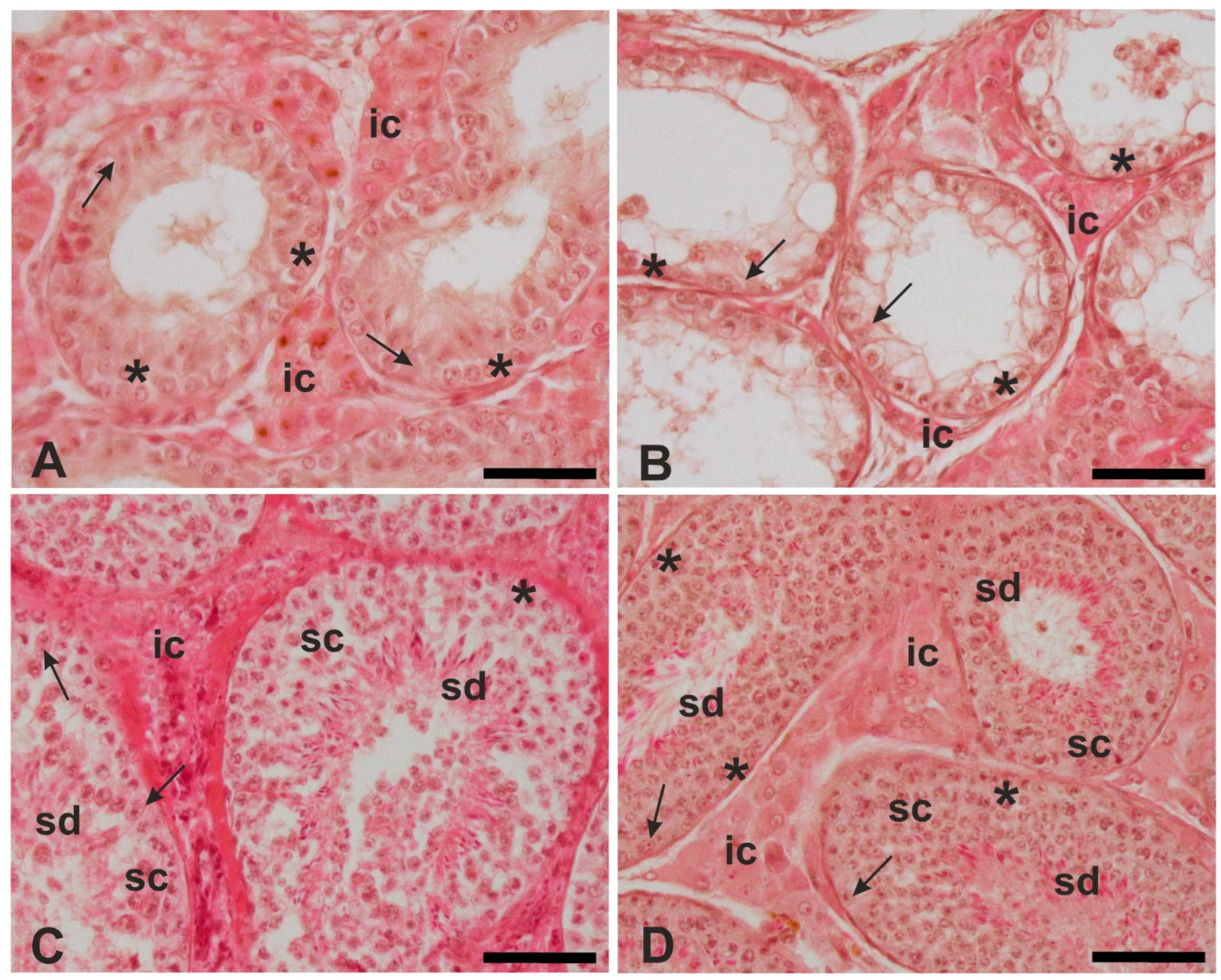
Light micrographs of the abdominal (**A**) and inguinal (**B**) retained testis; (**C**) contralateral scrotal testis of the abdominal/inguinal cryptorchid equines, and control (**D**). ic, interstitial (Leydig) cells; sc, spermatocytes; sd, spermatids; arrow, Sertoli cells; asterisk, spermatogonium. Hematoxylin–eosin staining. Scale bars: 50 μm.

**Figure 9:**
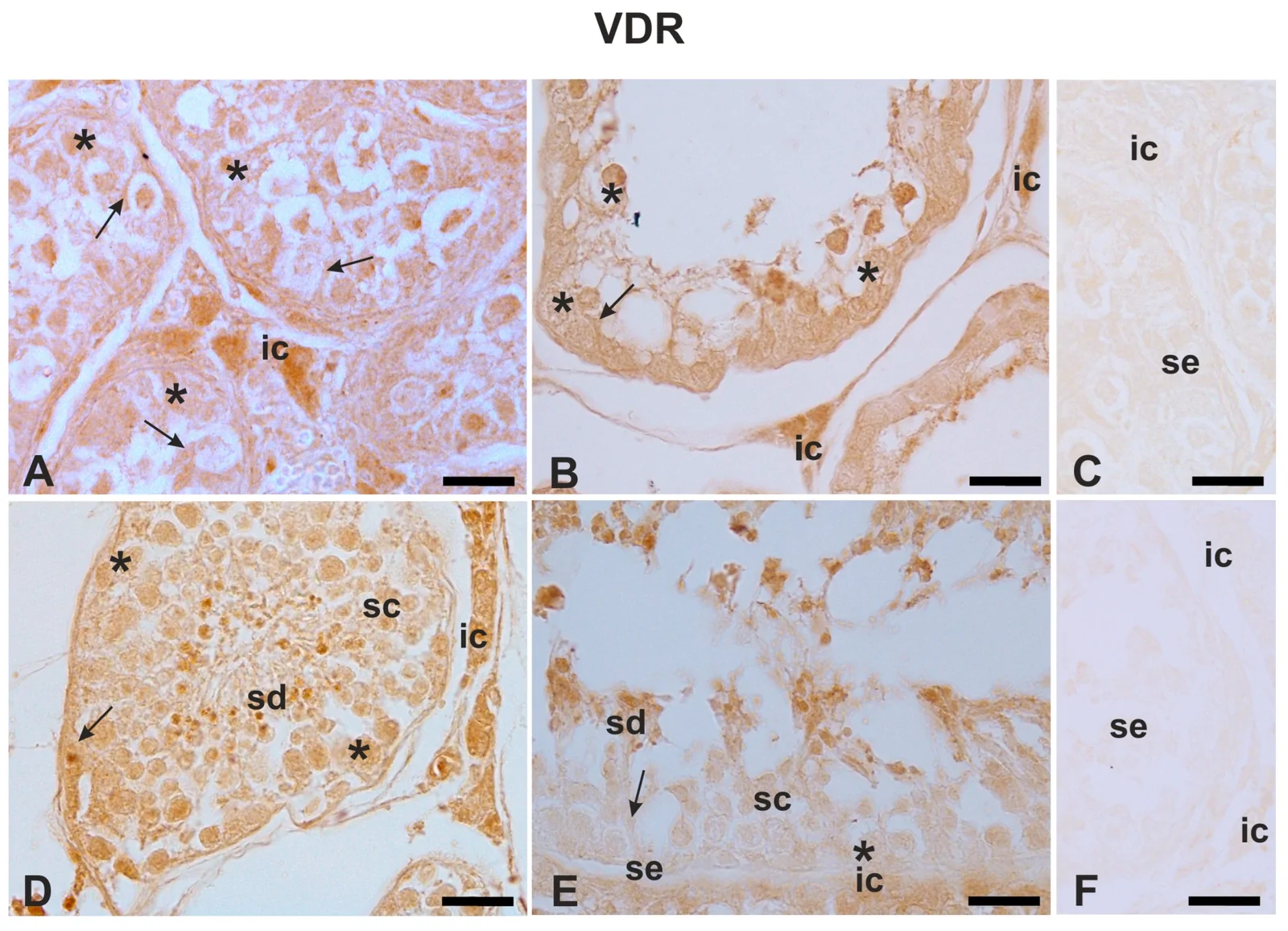
Localization of the VDR in the abdominal-retained testes (**A**), inguinal-retained testes (**B**), in scrotal testis from the abdominal/inguinal (**D**) and control (**E**) equines. (**C**,**F**) display the absence of immunoreactivity in negative controls in retained (**C**) and scrotal (**F**) testes. ic, interstitial (Leydig) cells; sc, spermatocytes; sd, spermatids; se, seminiferous epithelium; arrow, Sertoli cells; asterisk, spermatogonium. Scale bars: 20 μm.

**Figure 10:**
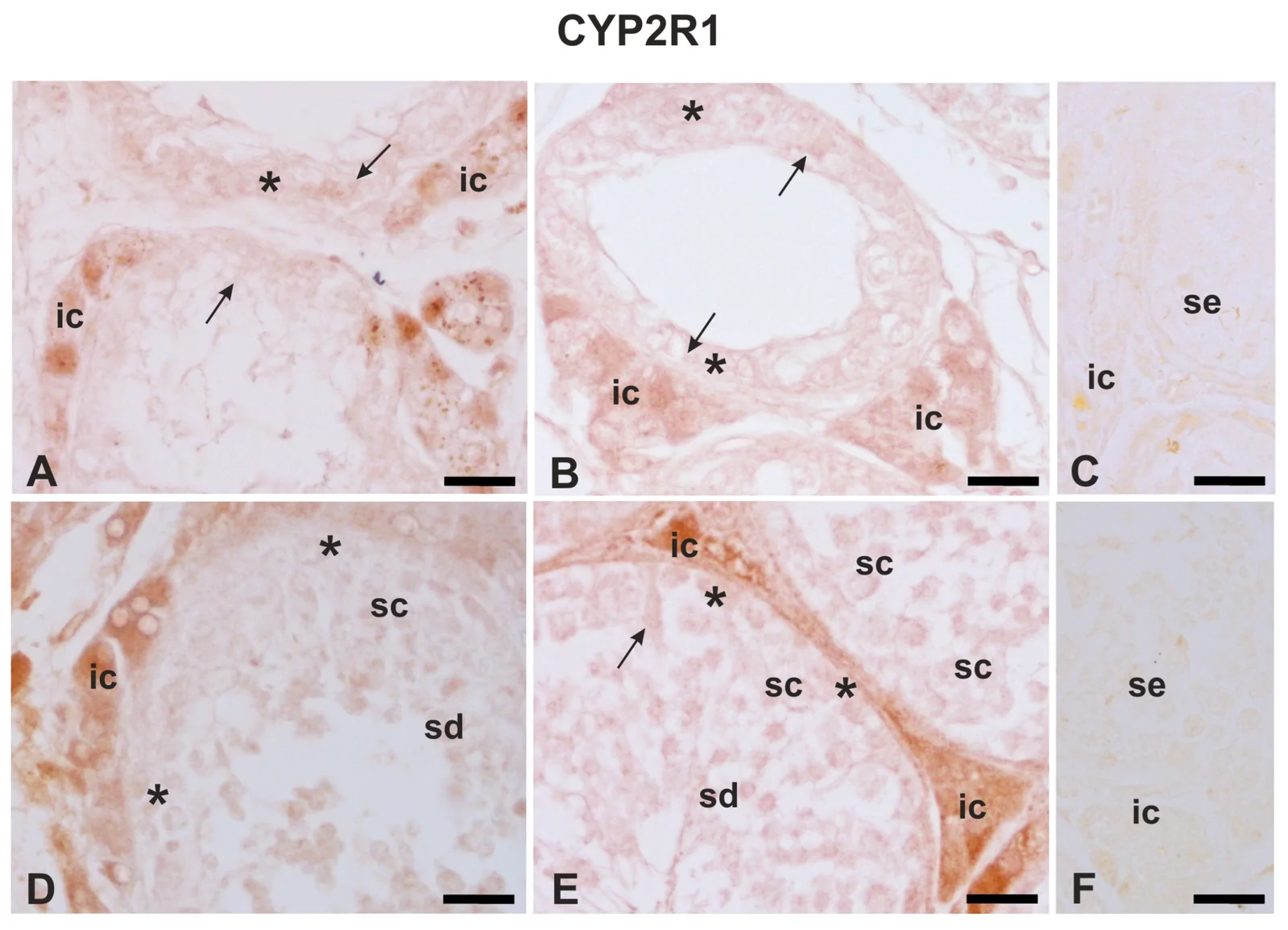
Localization of the CYP2R1 in the abdominal-retained testes (**A**), inguinal-retained testes (**B**), in scrotal testis from the abdominal/inguinal (**D**) and control (**E**) equines. (**C**,**F**) display the absence of immunoreactivity in negative controls in retained (**C**) and scrotal (**F**) testes. ic, interstitial (Leydig) cells; sc, spermatocytes; sd, spermatids; se, seminiferous epithelium; arrow, Sertoli cells; asterisk, spermatogonium. Scale bars: 20 μm.

**Figure 11:**
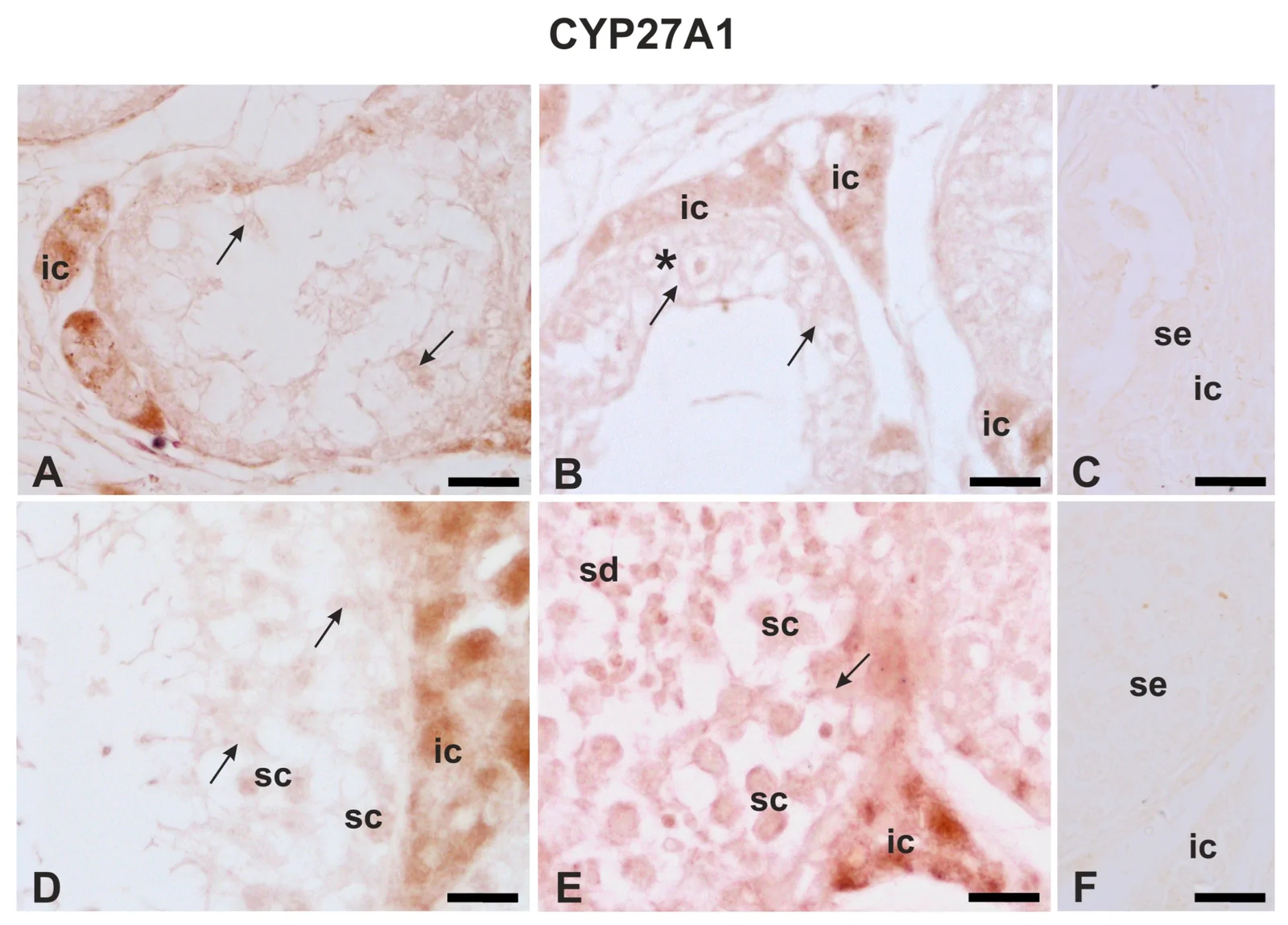
Localization of the CYP27A1 in the abdominal-retained testes (**A**), inguinal-retained testes (**B**), in scrotal testis from the abdominal/inguinal (**D**) and control (**E**) equines. (**C**,**F**) display the absence of immunoreactivity in negative controls in retained (**C**) and scrotal (**F**) testes. ic, interstitial (Leydig) cells; sc, spermatocytes; sd, spermatids; se, seminiferous epithelium; arrow, Sertoli cells; asterisk, spermatogonium. Scale bars: 20 μm.

**Figure 12:**
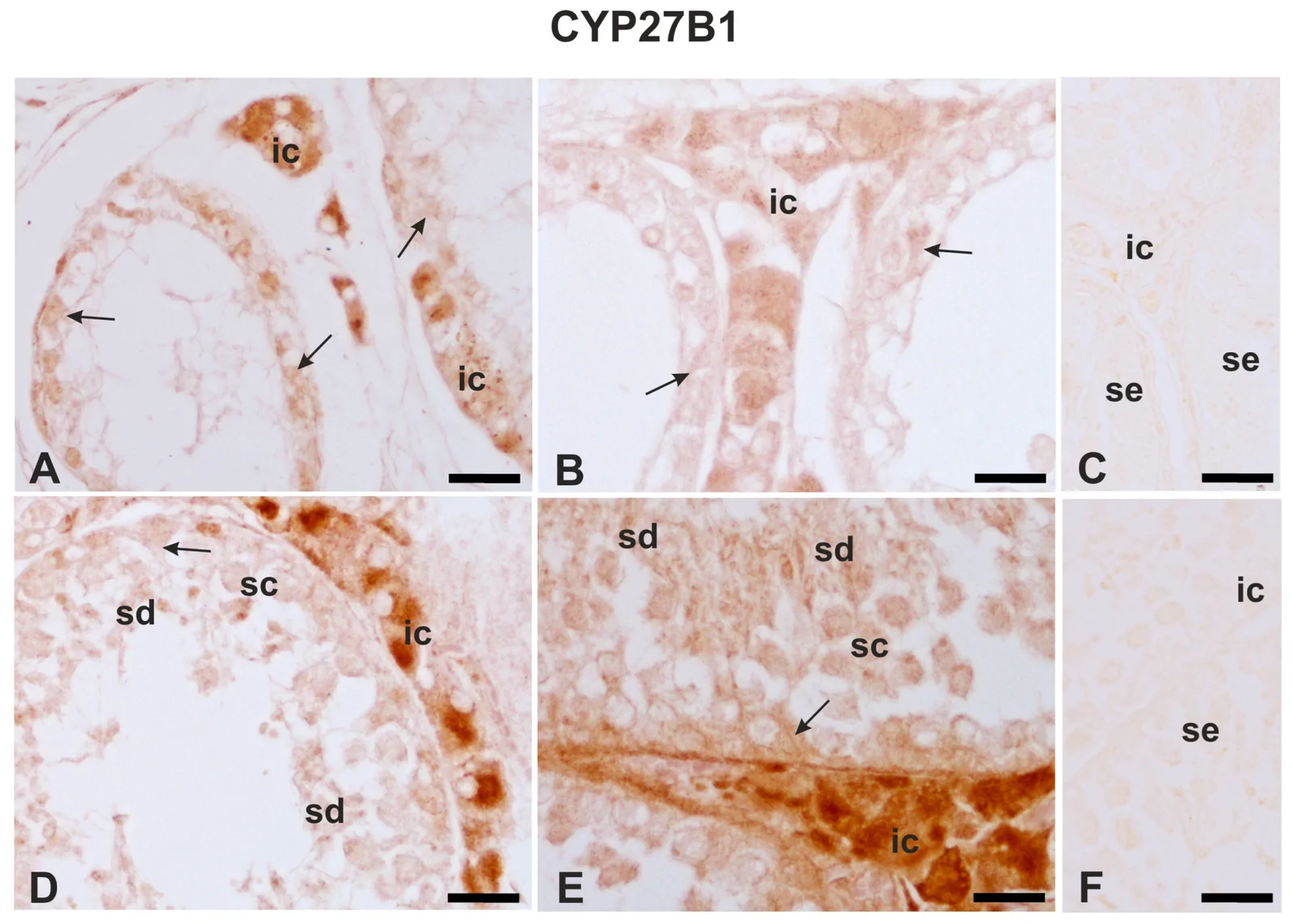
Localization of the CYP27B1 in the abdominal-retained testes (**A**), inguinal-retained testes (**B**), in scrotal testis from the abdominal/inguinal (**D**) and control (**E**) equines. (**C**,**F**) display the absence of immunoreactivity in negative controls in retained (**C**) and scrotal (**F**) testes. ic, interstitial (Leydig) cells; sc, spermatocytes; sd, spermatids; se, seminiferous epithelium; arrow, Sertoli cells. Scale bars: 20 μm.

**Table 1:** The table shows breed, age, and localization of the retained testicle of the equines enrolled in the study.

Subject	1	2	3	4	5	6	7	8	9	10	11	12	13	14	15	16	17	18
Breed	Q	S	S	M	M	M	M	S	Q	T	T	M	S	M	M	T	M	M
Age	3 y	8 y	2 y	6 y	3 y	3 y	4 y	4 y	4 y	3 y	3 y	5 y	4 y	5 y	5 y	3 y	4 y	3 y
Testicle retention	I	A	I	A	A	I	A	I	I	A	A	A	A	C	C	C	C	C

Breed: Q = quarter horse; S = Italian sadle, T = trotter; M = murgese. Testicle retention: A = abdominal; I = inguinal; C = control; Age: y = years.

**Table 2:** The table shows for each gene listed, the sequences of each couple of primers employed in the RT-PCR reactions and the expected size (bp) of the corresponding amplicon.

Gene	Oligo Name	Sequence (5′-3′)	Amplicon Size (bp)
VDR	VDR forward	AGTTCCGGCCTCCAGTTC	330
	VDR reverse	GTGAGGTCTCTGAACCCTG	
CYP2R1	CYP2R1 forward	TACTGGAGAGACCCAGAAAT	245
	CYP2R1 reverse	GTCTTTCTGCACAGATGAGG	
CYP27A1	CYP27A1 forward	CAAGAGGGCAAGTACCCAGT	325
	CYP27A1 reverse	GTAGGAAATAGCTTGGAAGGC	
CYP27B1	CYP27B1 forward	GCGAAGAATGGCAGAGGC	200
	CYP27B1 reverse	CCTTCTAGTCCAAACTTGTAAAA	
GAPDH	GAPDH forward	GTTTGTGATGGGCGTGAACC	254
	GAPDH reverse	TTGGCAGCACCAGTAGGAGC	

**Table 3:** Summary of the densitometric results of VDR, CYP2R1, CYP27A1, CYP27B1 protein expression in abdominal- and inguinal-retained testes, contralateral scrotal testes of cryptorchid subjects, and control testes.

	Protein Expression			
	VDR	CYP2R1	CYP27A1	CYP27B1
C	++	++	++	++
SA	++	+	++	+±
A	+	±	+	+
SI	+	+	+±	++
I	+	+	±	±
Not retained	+±	+	+±	+±
Retained	+	±	±	+

C, control testis; SA, contralateral scrotal testis of the abdominal-retained testicle; A, abdominal-retained testicle; SI, contralateral scrotal testis of the inguinal-retained testicle; I, inguinal-retained testicle; Not retained, sum of all not retained scrotal testicles; Retained, sum of all retained testicles. The level of protein expression was indicated by ++, (highest); +± (high); + (medium); ± (low).

**Table 4:** Expression of VDR, CYP2R1, CYP27A1, CYP27B1 in abdominal- and inguinal-retained testis, contralateral scrotal testis of cryptorchid subjects, and control testis.

Sample	VDR					CYP2R1					CYP27A1					CYP27B1				
	ic	Sc	g	spc	sd	ic	Sc	g	spc	sd	ic	Sc	g	spc	sd	ic	Sc	g	spc	sd
A	3	2	2	a	a	3	±	±	a	a	3	±	0	a	a	3	2	0	a	a
I	2	1	1	a	a	3	±	±	a	a	2	±	0/±	a	a	2	±	0	a	a
SA	3	±	1	1	3	3	±	±	±	1	3	±	0	±	0	3	±	0	±	2
SI	2	±	1	1	3	3	±	±	±	1	3	±	0	±	0	3	±	0	±	2
C	2	±	±	±	3	3	±	±	±	1	3	±	0	1	2	3	±	0	1	2

Samples: A, abdominal-retained testis; I, inguinal-retained testis; SA, scrotal testis of abdominal cryptorchid subjects; SI, scrotal testis of inguinal cryptorchid subjects; C, scrotal testis of control subjects. Cell type: g, spermatogonia; ic, interstitial cells, Sc, Sertoli cells; sd, spermatids. Staining: a, absent; 0, no staining; ±, faintly visible staining; 1, weak staining; 2, moderate staining; 3, strong staining.

## Data Availability

The data presented in this study are available on request from the corresponding author upon reasonable request.

## Abbreviations

The following abbreviations are used in this manuscript:

VD	Vitamin D
VDR	Vitamin D receptor
CYP27B1	A 1a-hydroxylase
CYP2R1	A 25-hydroxylase
CYP27A1	A 25-hydroxylase

## References

[1] Arighi M., McKinnon A.O., Squires E.I., Vaala W.E., Varner D.D. (2011). Testicular descent. Equine Reproduction.

[2] Gao Y., Wang C., Wang K., He C., Hu K., Liang M. (2022). The effects and molecular mechanism of heat stress on spermatogenesis and the mitigation measures. Syst. Biol. Reprod. Med..

[3] Amann R.P., Veeramachaneni D.N. (2007). Cryptorchidism in common eutherian mammals. Reproduction.

[4] Foresta C., Zuccarello D., Garolla A., Ferlin A. (2008). Role of hormones, genes, and environment in human cryptorchidism. Endocr. Rev..

[5] Blomberg-Jensen M., Gerner-Lawaetz J., Andersson A.M., Petersen J.H., Nordkap L., Bang A.K., Ekbom P., Joensen U.N., Prætorius L., Lundstrøm P. (2016). Vitamin D deficiency and low ionized calcium are linked with semen quality and sex steroid levels in infertile men. Hum. Reprod..

[6] Lombardo F., Sgrò P., Salacone P., Gilio B., Gandini L., Dondero F., Jannini E.A., Lenzi A. (2005). Androgens and fertility. J. Endocrinol. Investig..

[7] Chung J.J., Navarro B., Krapivinsky G., Krapivinsky L., Clapham D.E. (2011). A novel gene required for male fertility and functional CATSPER channel formation in spermatozoa. Nat. Commun..

[8] Garczynska E., Handelsman D.J. (1991). The role of calcium in follicle-stimulating hormone signal transduction in Sertoli cells. J. Biol. Chem..

[9] Yoshida M., Kawano N., Yoshida K. (2008). Control of sperm motility and fertility: Diverse factors and common mechanisms. Cell. Mol. Life Sci..

[10] Hejmej A., Bilinska B. (2008). The effects of chryptorchidism on the regulation of steroidogenesis and gap junctional communication in equine testes. Endokrynol. Pol..

[11] Cox J.E. (1989). Testosterone concentrations in normal and cryptorchid horses. Response to human chorionic gonadotrophin. Anim. Reprod. Sci..

[12] Bouillon R., Carmeliet G., Verlinden L., van Etten E., Verstuyf A., Luderer H.F., Lieben L., Mathieu C., Demay M. (2008). Vitamin D and human health: Lessons from vitamin D receptor null mice. Endocr. Rev..

[13] Dusso A.S., Brown A.J., Slatopolsky E. (2005). Vitamin D. Am. J. Physiol. Ren. Physiol..

[14] Jones G., Prosser D.E., Kaufmann M. (2014). Cytochrome P450-mediated metabolism of vitamin D. J. Lipid Res..

[15] Haussler M.R., Jurutka P.W., Mizwicki M., Norman A.W. (2011). Vitamin D receptor (VDR)-mediated actions of 1α,25(OH)_2_vitamin D_3_: Genomic and non-genomic mechanisms. Best Pract. Res. Clin. Endocrinol. Metab..

[16] Norman A.W. (2006). Minireview: Vitamin D receptor: New assignments for an already busy receptor. Endocrinology.

[17] Zhu J., DeLuca H.F. (2012). Vitamin D 25-hydroxylase—Four decades of searching, are we there yet?. Arch. Biochem. Biophys..

[18] Schryver H.F., Foose T.J., Williams J., Hintz H.F. (1983). Calcium excretion in feces of ungulates. Comp. Biochem. Physiol. A Comp. Physiol..

[19] Toribio R.E., Kohn C.W., Chew D.J., Sams R.A., Rosol T.J. (2001). Comparison of serum parathyroid hormone and ionized calcium and magnesium concentrations and fractional urinary clearance of calcium and phosphorus in healthy horses and horses with enterocolitis. Am. J. Vet. Res..

[20] Breidenbach A., Schlumbohm C., Harmeyer J. (1998). Peculiarities of vitamin D and of the calcium and phosphate homeostatic system in horses. Vet. Res..

[21] Blomberg-Jensen M., Nielsen J.E., Jørgensen A., Rajpert-De Meyts E., Kristensen D.M., Jørgensen N., Skakkebæk N.E., Juul A., Leffers H. (2010). Vitamin D receptor and vitamin D metabolizing enzymes are expressed in the human male reproductive tract. Hum. Reprod..

[22] Foresta C., Strapazzon G., De Toni L., Perilli L., Di Mambro A., Muciaccia B., Sartori L., Selice R. (2011). Bone mineral and testicular failure: Evidence for a role of vitamin D 25-hydroxylase in human testis. J. Clin. Endocrinol. Metab..

[23] Mahmoudi A.R., Zarnani A.H., Jeddi-Tehrani M., Katouzian L., Tavakoli M., Soltanghoraei H., Mirzadegan E. (2013). Distribution of vitamin D receptor and 1α-hydroxylase in male mouse reproductive tract. Reprod. Sci..

[24] Yao X., El-Samahy M.A., Yang H., Feng X., Li F., Meng F., Nie H., Wang F. (2018). Age-associated expression of vitamin D receptor and vitamin D metabolizing enzymes in the male reproductive tract and sperm of Hu sheep. Anim. Reprod. Sci..

[25] Rouge M., Elkhatib R., Delalande C., Cognie J., Reigner F., Barriere P., Deleuze S., Coutsy M., Legendre F., Galera P. (2022). Investigation of equine testis contribution to vitamin D bioactivation. Domest. Anim. Endocrinol..

[26] Vuolo L., Di Somma C., Faggiano A., Colao A. (2012). Vitamin D and cancer. Trends Endocrinol. Metab..

[27] Scott C.J., Christensen B.W., Dechant J.E., Espinosa P., LaDouceur E.E. (2015). Theriogenology question of the month. J. Am. Vet. Med. Assoc..

[28] Cinone M., Albrizio M., Guaricci A.C., Lacitignola L., Desantis S. (2024). Testicular expression of heat shock proteins 60, 70, and 90 in cryptorchid horses. Theriogenology.

[29] Schorra P., Stokesb C.S., Volmer D.A. (2023). Improved quantitative LC-MS/MS analysis of vitamin D metabolites in serum after one-pot double derivatization. J. Pharm. Biomed. Anal..

[30] Albrizio M., Roscino M.T., Trisolini C., Binetti F., Rizzo A., Sciorsci R.L. (2013). The expression of the leptin receptor in the ovary of the queen. Leptin receptor expression in queen ovary. Res. Vet. Sci..

[31] Mannuel H.D., Mitikiri N., Khan M., Hussain A. (2012). Testicular germ cell tumors: Biology and clinical update. Curr. Opin. Oncol..

[32] Soto-Heras S., Reinacher L., Wang B., Oh J.E., Bunnell M., Park C.J., Hess R.A., Ko C.J. (2024). Cryptorchidism and testicular cancer in the dog: Unresolved questions and challenges in translating insights from human studies. Biol. Reprod..

[33] Peterson D.E. (1984). Equine testicular tumors. J. Equine Vet. Sci..

[34] Maalouf N.M. (2008). The non-calciotropic actions of vitamin D: Recent clinical developments. Curr. Opin. Nephrol. Hypertens..

[35] Sonnenberg J., Luine V.N., Krey L.C., Christakos S. (1986). 1,25-Dihydroxyvitamin D_3_ treatment results in increased choline acetyltransferase activity in specific brain nuclei. Endocrinology.

[36] Inpanbutr N., Reiswig J.D., Bacon W.L., Slemons R.D., Iacopino A.M. (1996). Effect of vitamin D on testicular CaBP28K expression and serum testosterone in chickens. Biol. Reprod..

[37] Azarpeykan S., Dittmer K.E., Marshall J.C., Perera K.C., Gee E.K., Acke E., Firth E.C. (2016). Evaluation and comparison of vitamin D responsive gene expression in ovine, canine and equine kidney. PLoS ONE.

[38] Morris H.A., Anderson P.H. (2010). Autocrine and paracrine actions of vitamin D. Clin. Biochem. Rev..

[39] Wang X., Liu Q., Zhuang Z., Cheng J., Zhang W., Jiang Q., Guo Y., Li R., Lu X., Cui L. (2024). Decoding the pathogenesis of spermatogenic failure in cryptorchidism through single-cell transcriptomic profiling. Cell Rep. Med..

[40] Stachowiak M., Nowacka-Woszuk J., Szabelska-Beresewicz A., Zyprych-Walczak J., Krzemińska P., Sosiński O., Nowak T., Świtoński M. (2024). A massive alteration of gene expression in undescended testicles of dogs and the association of KAT6A variants with cryptorchidism. Proc. Natl. Acad. Sci. USA.

[41] Merke J., Hügel U., Ritz E. (1985). Nuclear testicular 1,25-dihydroxyvitamin D_3_ receptors in Sertoli cells and seminiferous tubules of adult rodents. Biochem. Biophys. Res. Commun..

[42] Hirai T., Tsujimura A., Ueda T., Fujita K., Matsuoka Y., Takao T., Okuyama A., Nonomura N. (2009). Effect of 1,25-dihydroxyvitamin D on testicular morphology and gene expression in experimental cryptorchid mouse: Testis specific cDNA microarray analysis and potential implication in male infertility. J. Urol..

[43] Johnson J.A., Grande J.P., Roche P.C., Kumar R. (1996). Immunohistochemical detection and distribution of the 1,25-dihydroxyvitamin D_3_ receptor in rat reproductive tissues. Histochem. Cell Biol..

[44] Oliveira A.G., Dornas R.A., Kalapothakis E., Hess R.A., Mahecha G.A., Oliveira C.A. (2013). Vitamin D_3_ and androstenone regulation in the equine reproductive tract. Theriogenology.

[45] La Vignera S., Condorelli R.A., Cimino L., Russo G.I., Morgia G., Calogero A.E. (2016). Late-onset hypogonadism: The advantages of treatment with human chorionic gonadotropin rather than testosterone. Aging Male.

[46] Nangia A.K., Hill O., Waterman M.D., Schwender C.E.B., Memoli V. (2007). Testicular maturation arrest to testis cancer: Spectrum of expression of the vitamin D receptor and vitamin D treatment in vitro. J. Urol..

[47] Craig T.A., Sommer S., Sussman C.R., Grande J.P., Kumar R. (2008). Expression and regulation of the vitamin D receptor in the zebrafish, Danio rerio. J. Bone Miner. Res..

[48] Akerstrom V.L., Walters M.R. (1992). Physiological effects of 1,25-dihydroxyvitamin D_3_ in TM4 Sertoli cell line. Am. J. Physiol..

[49] Uhland A.M., Kwiecinski G.G., DeLuca H.F. (1992). Normalization of serum calcium restores fertility in vitamin D-deficient male rats. J. Nutr..

[50] Menegaz D., Rosso A., Royer C., Leite L.D., Santos A.R., Silva F.R. (2009). Role of 1alpha,25(OH)_2_ vitamin D_3_ on alpha-[1-^14^C]MeAIB accumulation in immature rat testis. Steroids.

[51] de Angelis C., Galdiero M., Pivonello C., Garifalos F., Menafra D., Cariati F., Salzano C., Galdiero G., Piscopo M., Vece A. (2017). The role of vitamin D in male fertility: A focus on the testis. Rev. Endocr. Metab. Disord..

